# Hybrid pneumatic-hydraulic actuation for MRI-guided robotic stereotactic neurointervention

**DOI:** 10.1126/sciadv.ady3624

**Published:** 2025-09-03

**Authors:** Shaoping Huang, Zhao He, Yi Chen, Jiafan Chen, Shijie Hong, Yi Zhang, Longyu Xu, Yixin Pan, Shuo Ma, Lian Xuan, Qingdang Meng, Yong Yang, Yangyang Xu, Zecai Lin, Chuqian Lou, Cheng Zhou, Weidong Chen, Bomin Sun, Qingfang Sun, Yuan Feng, Anzhu Gao, Guang-Zhong Yang

**Affiliations:** ^1^ Shanghai Key Laboratory of Flexible Medical Robotics, Tongren Hospital, Institute of Medical Robotics, Shanghai Jiao Tong University, Shanghai 200336, China.; ^2^ Institute of Medical Robotics, School of Biomedical Engineering, Shanghai Jiao Tong University, Shanghai 200030, China.; ^3^ National Engineering Research Center of Advanced Magnetic Resonance Technologies for Diagnosis and Therapy (NERC-AMRT), School of Biomedical Engineering, Shanghai Jiao Tong University, Shanghai 200240, China.; ^4^ Department of Neurosurgery, Ruijin Hospital affiliated to Shanghai Jiao Tong University School of Medicine, Shanghai 200025, China.; ^5^ Institute of Medical Robotics, School of Automation and Intelligent Sensing, Shanghai Jiao Tong University, Shanghai 200240, China.; ^6^ Materials and Technology Center of Robotics, Empa, Dübendorf 8600, Switzerland.; ^7^ Department of Radiology, Ruijin Hospital affiliated to Shanghai Jiao Tong University School of Medicine, Shanghai 200025, China.

## Abstract

Stereotactic neurointervention is a common procedure for biopsy, injection, ablation, and implantation of electrodes for deep brain stimulation. Guided by preoperative imaging, conventional approaches are mostly performed manually, lacking operation stability and interactive feedback. The intraoperative magnetic resonance imaging (MRI) guidance enables both structural and functional assessment during operation, permitting interactive adaptation to tissue deformation and avoidance of critical anatomical regions. Here, we report an MRI-guided robotic system for stereotactic neurointervention. A macro-micro hybrid pneumatic-hydraulic actuated stereotactic robot with a large range of motion and high precision is developed. This is coupled with a compact bioinspired soft actuator for target intervention. A global-focal MRI sequence is proposed for interactive navigation, closed-loop control, and precise targeting. Validation is performed with phantom, cadaveric, and in vivo animal studies, showing positional accuracies of 0.39, 0.68, and 0.14 millimeters, respectively, demonstrating superior performance compared to the current state of the art in robotic-assisted stereotactic neurointervention.

## Teaser

An MRI-guided hybrid pneumatic-hydraulic robot with bioinspired needle actuator achieves submillimeter stereotactic accuracy.

## INTRODUCTION

Neurological disorders are the second leading cause of death globally (*1*), where survivors require extensive resources for long-term therapy and rehabilitation. Stereotactic neurointervention is widely used for procedures such as biopsy, resection, injection, ablation, stereoelectroencephalography, and implantation of electrodes for deep brain stimulation (DBS) (*2*). Magnetic resonance imaging (MRI) is an effective tool for preoperative planning due to its versatility in providing high-contrast soft-tissue characterization and functional information, including perfusion, diffusion, and accurate temperature mapping without ionizing radiation (*3*). Intraoperatively, traditional stereotactic neurointervention is challenging to perform under MRI guidance due to restricted operational space and MR-incompatible hardware that may cause signal loss, field distortion, and degradation of signal-to-noise ratio (SNR) (*4*). A standard stereotactic surgical procedure encompasses three main stages (*2*). First, preoperative imaging is performed using MRI and/or computed tomography (CT) to obtain anatomical details of the brain. Second, a preferred surgical path is carefully planned in accordance with the procedural requirements while avoiding critical structures and blood vessels. The resultant trajectory is then mapped to adjustable parameters of the stereotactic frame via registration. Once validated, a burr hole is drilled, and the dura mater is subsequently punctured using a guide mounted on the frame. The intervention device is then aligned and inserted into the designated target for subsequent procedures, whether for ablation, biopsy, cyst aspiration, implantation, or drug delivery.

Despite being widely adopted clinically, stereotactic neurointervention continues to encounter challenges due to the high precision (submillimeter) (*5*) required for intervention, as well as limited manual operation and a lack of intraoperative imaging feedback. Errors include registration inaccuracy between the stereotactic frame and the imaging volume, as well as intraoperative vibrations induced by craniotomy procedures and brain shift due to the release of intracranial pressure and cerebrospinal fluid loss after craniotomy. These are further coupled with processing and assembly inaccuracy of the mechanical system, which can reach up to 5 mm in many cases (*6*), potentially resulting in treatment failure, functional zone injury, or cerebral hemorrhage. To address the challenges of manual neurosurgical interventions, robotic platforms such as Neuromate (*7*) and ROSA (*8*) have gained widespread clinical adoption for neurological procedures, demonstrating the notable clinical value of robotics-assisted neurointervention. Nevertheless, their reliance on preoperative CT-based or optical navigation fundamentally constrains clinical efficacy. This dependency results in some critical intraoperative limitations, such as the inability to achieve interactive visualization of neuroanatomical structures during instrument advancement and the absence of quantitative thermographic surveillance during ablation therapy, ultimately compromising therapeutic precision in functional neurosurgery.

The value of intraoperative MRI has long been appreciated for addressing the above limitations. It allows for patients to be scanned continuously during intervention to verify the position and anatomy of the brain, ensuring that the instrument is aligned with the treatment plan. Several systems have been designed for stereotactic neurointervention under intraoperative MRI guidance. Representative commercial products include Leksell Vantage Frame (Elekta AB) (*5*), AXiiiS Stereotactic Miniframe (Monteris Medical) (*9*), NexFrame (Medtronic) (*10*, *11*), and SmartFrame (ClearPoint Neuro) (*12*, *13*). These devices aim to enhance intervention accuracy and reproducibility, alleviate patient discomfort, and improve MR compatibility. The procedure, however, remains hand driven, and it is challenging to operate manually within the small bore of MRI, especially when performing fine instrument adjustment or needle insertion. Furthermore, procedural success remains operator dependent, limiting reproducibility across skill levels. To address these challenges, several MR-safe or MR-conditional (*4*) robotic systems have been developed for stereotactic neurointervention. The first Food and Drug Administration–approved MR-compatible robot for brain surgery was SYMBIS/NeuroArm (IMRIS) (*14*). This system contained two classic serial robotic arms, allowing for repeated checks of the current anatomical status through a movable MRI magnet for intraoperative imaging. It offered specific advantages for intricate craniotomy procedures, such as glioma resection (*15*). The NeuroBlate system (Monteris Medical) (*16*) had 2 df and could perform closed-loop robotic procedures, such as laser interstitial thermal therapy (LITT) and drug delivery under MRI guidance. However, it relied on a stereotactic system to enable on-trajectory deployment of the Mini-Bolt (*16*) into the skull. Extensive research had also been directed to addressing associated challenges related to actuation, sensing, materials, MR-compatible interventional instruments designs, and interactive/real-time MRI (*17*, *18*). For example, Fischer and colleagues (*19*, *20*) developed an 8-df MR-conditional stereotactic robotic system on the basis of piezoelectric actuation. Interference of MRI images caused by the piezoelectric motors has been addressed, and the tip positioning accuracy of the system could reach 1.45 ± 0.66 mm. A 3-df MR-safe needle-positioning robot (*21*, *22*), actuated by pneumatic motors and mounted on a 3-df manual adjustment system, has been proposed by Stoianovici *et al.* The achieved absolute accuracy guided by intraoperative MRI was 1.55 ± 0.81 mm (*22*). Kwok and colleagues demonstrated a hydraulic frameless stereotactic system (*23*, *24*), achieving an accuracy of 1.7 mm on skull phantoms and 2.2 mm on a human cadaver. Real-time imaging is another bottleneck for interactive intraoperative guidance. A previous work has tackled the issue of MRI acquisition speed by applying techniques such as undersampling and accelerated reconstruction. Zufiria *et al.* (*25*), for example, presented a feature-based convolutional neural network (FbCNN) for interventional MRI reconstruction, achieving two-dimensional (2D) imaging within 0.5 s. Our previous work (*26*) introduced a deep unrolled neural network (LSFP-Net) for MRI-guided brain intervention with an acquisition window of 80/732.8 ms and an overall reconstruction latency of 0.4/3.66 s, for 2D and 3D imaging, respectively. Fast MRI-tracking methods with high temporal resolution have also been proposed, such as using 1D projection imaging with radio frequency (rf) markers (*24*, *27*) or using magnetic markers (*28*). Despite these advances, no robotic stereotactic neurointervention systems can thus far achieve fully-actuated stereotactic positioning and perform closed-loop intervention under interactive MRI with submillimeter accuracy.

Current challenges that hinder the accuracy of robotic neurointervention systems primarily stem from the following factors. First, the required df, large workspace, and deep insertion of the instrument tip impose inherently contradicting constraints for high-precision robot control (*2*). Second, interventional devices often necessitate long-distance insertion and rotational manipulation for procedures such as biopsy and directional ablation therapy (*16*), and developing a compact needle insertion mechanism with simultaneous rotation and translation for high-precision target intervention in a confined space is challenging. Third, balancing temporal and spatial resolution is a major difficulty for intraoperative MRI feedback (*26*). In addition, the strong magnetic fields and restrictions of the MR environment, combined with the high sensitivity (*29*) of MR signals, limit the materials, actuation methods, and sensing approaches that can be used (*4*).

**Fig. 1.**
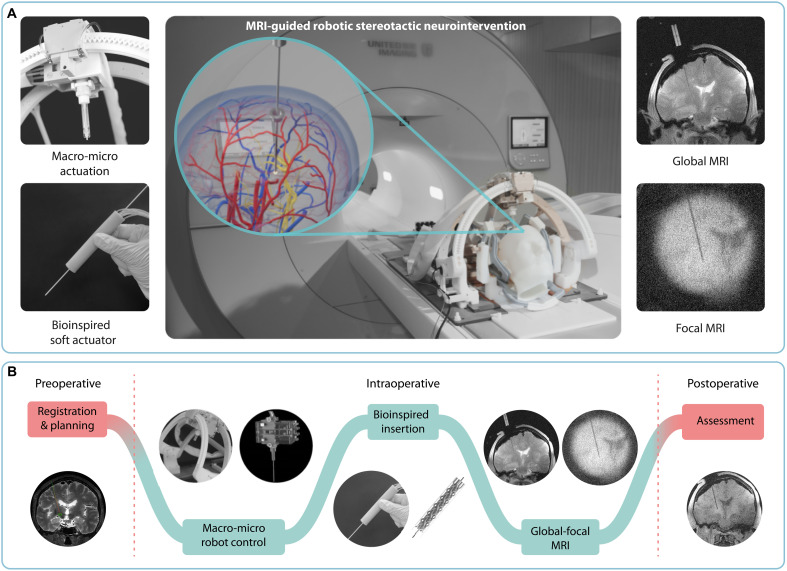
Illustration of the MRI-guided robotic stereotactic neurointervention system and its workflow. (**A**) The proposed MRI-guided robotic stereotactic neurointervention system consists of a macro-micro robot with hybrid pneumatic-hydraulic actuation and a bioinspired soft actuator guided by global-focal MRI. (**B**) Overall workflow of the robotic stereotactic neurointervention used in this study. In the preoperative stage, only one MRI scan is required, followed by registration between the MRI and the robot for preoperative planning. During operation, the robot can automatically move along the planned trajectory with high efficiency (fast pneumatic actuation for macropositioning) and accuracy (accurate hydraulic actuation for fine adjustment) guided by 3D MRI. A burr hole is drilled on the basis of the position on the cranial bone indicated by the robot, and a second anatomical MRI scan is performed to verify the error and tissue deformation caused by “brain shift,” such that further fine adjustments can be made. Once completed, the needle mounted on the bioinspired soft actuator is inserted while the MRI switches from the global mode to local mode for online monitoring of the instrument trajectory. The path and distance between the needle tip and the target are continuously calculated.

In this study, we propose a macro-micro stereotactic robot with high precision and a large range of motion, integrated with a compact bioinspired soft actuator that uses a global-focal MRI sequence for interactive navigation and precise target intervention (Fig. 1A, fig. S1, and movie S1). A hybrid pneumatic-hydraulic actuation stereotactic system is designed, which includes 4-df fast pneumatic macroactuation for global positioning and 4-df hydraulic microactuation for precise local adjustment. A bioinspired soft actuator is fabricated for target intervention, with features including compactness (Φ18.4 mm, 30 g), ease of manufacturing (3D printing), stability (locking force: 13 N), and cost-effectiveness (~$5). Its biomimetic peristaltic motion allows for precise and stable needle insertion. The global-focal MRI sequence allows for interactive navigation and closed-loop control during intervention (global MRI) and precise identification of the approaching target (focal MRI) as shown in Fig. 1B. Detailed functional experiments have been carried out to validate the performance of the system with phantom, cadaveric, and in vivo animal studies, and the relative merits of the proposed system compared to the current state of the art are summarized in table S1.

## RESULTS

### Hybrid pneumatic-hydraulic actuation with macro-micro motion

**Fig. 2.**
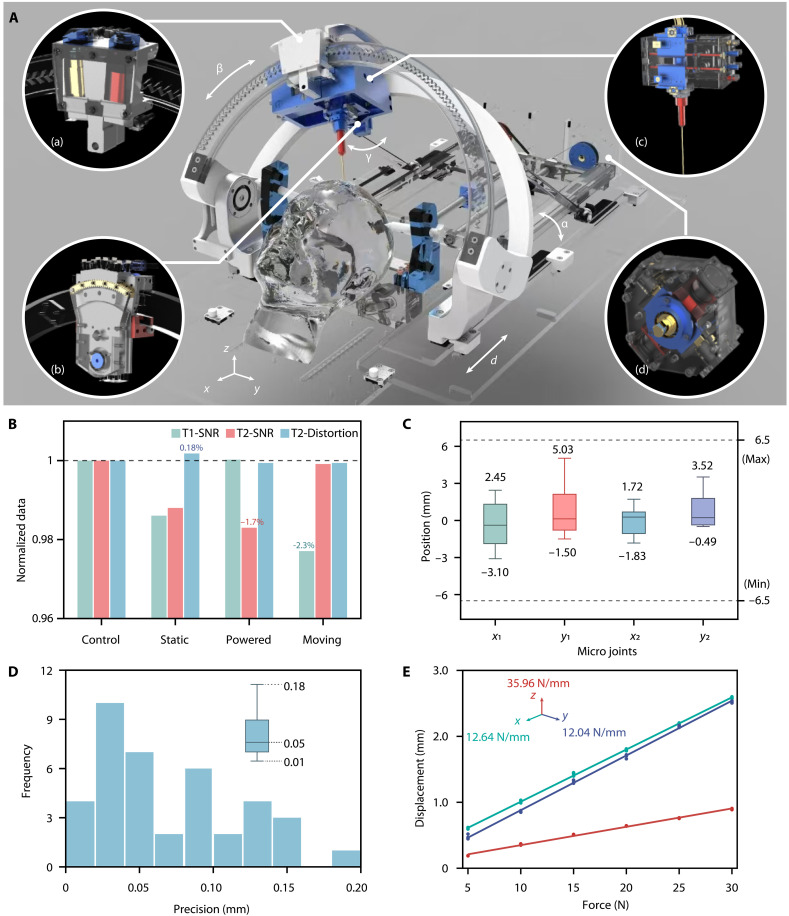
Design and performance evaluation of the macro-micro hybrid pneumatic-hydraulic actuated stereotactic robot. (**A**) Hybrid pneumatic-hydraulic actuated stereotactic robot. (a), (b), and (c) are used for pneumatic macroactuation, whereas (d) is responsible for linear translation (first df, ±50 mm) and lifting motion (second df, −65° to 20°) through a lead screw. (a) and (b) are conformally integrated on the front and back of the arc, respectively. (a) provides a large arc motion (third df, ±60°), whereas (b) enables rotation on the arc (fourth df, ±20°). (c) provides the microhydraulic actuation which is mounted on the macroactuation between (a) and (b). (**B**) Detailed MR compatibility tests (SNR_loss_ and distortion) were conducted, and comparisons were made between the control group and different robot operation stages: Static indicates the robot positioned adjacent to the phantom without power supply; powered represents the powered robot maintaining stationary position; and moving represents the robot executing programmed movements. (**C**) The fine adjustment results of four microhydraulic joints (*x*_1_, *y*_1_, *x*_2_, *y*_2_) based on 16 stereotactic experiments. (**D**) Histogram of tip error distribution for 39 repeatability tests with a 100-mm insertion length. (**E**) Force-displacement curves of the robot tip under 5- to 30-N loads (spaced by 5 N), with each test repeated four times and analyzed using polynomial fitting to evaluate the inherent stiffness of the robot through linear approximation.

The details of the macro-micro hybrid pneumatic-hydraulic actuation scheme for fast global positioning and high-precision local adjustment during stereotactic neurointervention are shown in Fig. 2A. The framework consists of a 4-df pneumatic macroactuation module (MAM) that meets the workspace requirement of stereotactic neurointervention and a 4-df hydraulic microactuation module (MiAM) for accurate fine adjustment after global positioning. The configuration of the MAM follows the classic Leksell frame (*30*), which includes translation (first df), lifting motion (second df), arc motion (third df), and rotation on the arc (fourth df) (fig. S2). Three actuators [Fig. 2A, (a), (b), and (d)] based on step pneumatic actuation (fig. S3) were designed for MAM. The ranges of MAM include the following: the first df (*d*) translation of ±50 mm, the second df (α) lifting motion from −65° to 20°, the third df (β) arc motion of ±60°, and the fourth df (γ) rotational motion on the arc of ±20° (Fig. 2A and fig. S2A). The forward and inverse kinematics of the MAM were established on the basis of its serial structure. Monte Carlo simulations (*31*) were used to map the workspace, and the results showed that MAM can fully cover a normal human brain (fig. S2B). We designed the 4-df MiAM to be a dual-plane hydraulically actuated (fig. S4) parallel mechanism (fig. S5 and movie S2) that allows for spatial attitude adjustment by moving the positions of the upper and lower planes in two orthogonal (*x*, *y*) axes. The planar workspace of MiAM (*x*, *y* = ±6.5 mm) is designed on the basis of the maximum error of the macroactuation. This error was simulated on the basis of the kinematics of MAM and the overall dimensions of the system for a typical 3-T MR bore of 600 mm. Extensive MR compatibility tests (SNR_loss_ and geometric distortion) were conducted for the robot. Comparisons were made between the control setting and different robot operation stages. Results showed that the robot demonstrated a maximum SNR_loss_ of −2.3% and maximum distortion of 0.18% (Fig. 2B and fig. S6). In 16 stereotactic experiments, we tested the compensation capabilities between microadjustment and macropositioning. The MAM achieved SE(3) pose tracking with positioning error < 5 mm and angular error < 1° through inverse kinematics and iterative Jacobian optimization (fig. S7). The MiAM performed the subsequent fine adjustment based on the kinematics with a maximum joint motion of 5.03 mm (<6.5 mm) to compensate for macroerrors (Fig. 2C). In the repeatability accuracy test, the average error was 0.05 mm and the maximum absolute error was 0.18 mm (*N* = 39) (Fig. 2D and movie S4). The system stiffness was measured as 12.64 N/mm (*x*), 12.04 N/mm (*y*), and 35.96 N/mm (*z*) (Fig. 2E).

### Bioinspired soft actuator for needle insertion

**Fig. 3.**
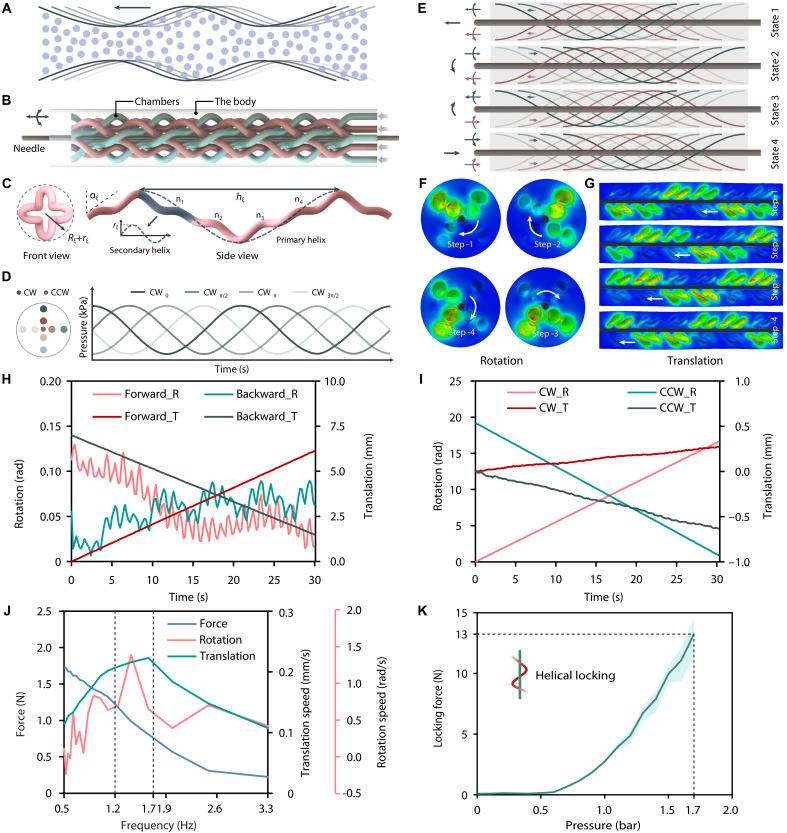
Design and performance characterization of the bioinspired soft actuator. (**A**) Illustration of the concept of peristaltic motion driven by traveling waves. (**B**) Structure of the bioinspired soft actuator with 2-df (translation and rotation). (**C**) Schematic of one chamber of the soft actuator. The helix has a diameter of *R*+*r*, where φ indicates the helical angle, λ indicates the length of one period of the chamber, and n_1_ to n_4_ indicate the position of the secondary helixes. (**D**) Left: Two pairs of pneumatically driven chambers. Right: Pressure variations in the four outer (CW) chambers. Each chamber is driven by a sinusoidal curve, with phase differences of 90°. (**E**) Four $C_{2}^{1}\times C_{2}^{1}$ types of motion based on different traveling wave sequences: forward translation, backward translation, CW rotation, and CCW rotation. (**F** and **G**) FEA strain result for states 2 and 4 of (E), showing four time steps forming one complete cycle. (**H**) Decoupled translation test results. Forward_R and Forward_T indicate the rotation and the translation during the forward motion test, respectively. Backward_R and Backward _T indicate the rotation and the translation during the backward motion test, respectively. (**I**) Decoupled rotation test results. CW_R and CW_T indicate the rotation and the translation during the CW rotation test, respectively. CW_R and CW_T indicate the rotation and the translation during the CCW rotation test, respectively. (**J**) Results of the decoupled translational and rotational speed and the corresponding insertion force during translation in different chamber frequencies (0.5 to 3.3 Hz). (**K**) Locking force of the needle with helical locking. The graph shows the means ± SD of *N* = 4 measurements at each data point.

The soft actuator was inspired by the peristaltic locomotion (Fig. 3A and fig. S8A) in biological systems (*32*). It emulated the peristaltic locomotion and extended this to helical wave propagation. This compact bioinspired soft actuator (Fig. 3B) features eight second-order helical chambers, enabling independent control of needle rotation and translation over extended insertion distances. The principle of traveling wave motion was shown in fig. S8. The helical chambers (Fig. 3C) provide a large contact area on the needle, ensuring stable synchronized rotation and translation of the actuator. The Materials and Methods section elaborated on the geometric modeling of helical chambers. Figure S9 illustrated the detailed analysis of helical morphology. By reversing the helical handedness of the chamber, the output translational direction is inverted without altering the rotational orientation. To resolve this spatial conflict while maintaining coaxial alignment, a secondary helix was introduced to structurally interleave the clockwise (CW) and counterclockwise (CCW) helical chambers (fig. S9E). Consequently, the chambers feature multiple secondary helices nested within the primary helix. On the basis of this structure, a set of traveling wave input motion enables synchronized rotation and translation in either CW or CCW chambers (Fig. 3D). By altering the propagation direction of the traveling waves, four ( $C_{2}^{1}\times C_{2}^{1}$ ) types of motion can be generated (Fig. 3E). The soft actuator was characterized using finite element analysis (FEA) using Abaqus for the strain field distributions of the helical chambers under decoupled rotation (Fig. 3F) and translation (Fig. 3G). Displacement and rotational motion of the needle in rotational and translational mode demonstrated the actuator’s decoupling capability in Abaqus (fig. S10). The analyses were extended to the strain variations at different cross-sectional positions and the traveling contact stress distribution on the surface of the needle (figs. S11 and S12 and movie S3). The soft actuator was fabricated via soft-material 3D printing (P400UHD, RAYSHAPE), with a Shore A hardness 30 A, a mass of 30 g, and dimensions of 18.4 mm (outer diameter) by 105 mm (length). Its internal structure and fabrication quality were analyzed through tomographic reconstruction (20 μm slice thickness) using Micro-CT (Xradia 520 Versa), as shown in fig. S13. Figure S14 illustrates the methodology for assembling the soft actuator onto the MiAM. The inlets of the soft actuator were bonded to eight silicone tubes using adhesive (S-8005X, ALDERS), with the opposite tube termini connected to the pressure control system (fig. S15). The decoupled motion (movie S3) and load capacity were characterized using a high-precision camera (ARAMIS 3D Camera, Gom) and a microforce sensor (Nano-17, ATI). A pressure controller (MK4+, Elveflow, Elvesys) was used for pneumatic pressure regulation of different chambers. In the decoupled translation test (Fig. 3H), forward and backward displacements were 6.13 and 5.51 mm, with rotations of 0.12 and 0.08 rad, respectively. In the decoupled rotation test (Fig. 3I), CW and CCW rotations were 16.65 and 18.31 rad, with translations of 0.27 and 0.63 mm, respectively. The decoupled translational speed, rotational speed, and insertion force in the translational state were tested under different traveling wave frequencies (Fig. 3J). Within the range of 1.2 to 1.7 Hz, the actuator achieved a translational speed of >0.2 mm/s and an insertion force of >0.5 N. The maximum locking force was 13 N at 1.7 bar, which was measured by applying static pressure to a single chamber (Fig. 3K).

### Interactive global-focal MRI

**Fig. 4.**
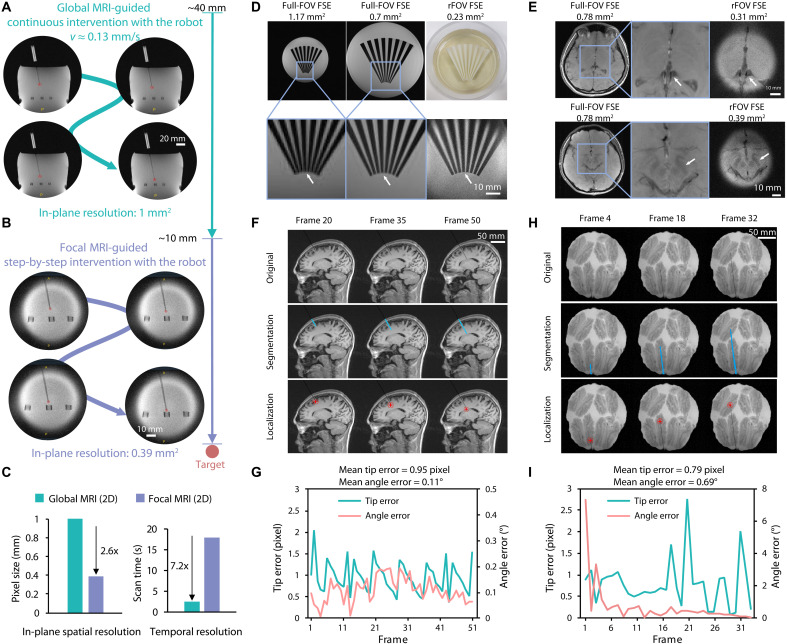
Interactive global-focal MRI. (**A**) Global MRI-guided continuous intervention with the robot. The in-plane resolution of global MRI is 1 mm^2^. (**B**) Focal MRI-guided step-by-step intervention with the robot. The in-plane resolution of focal MRI is 0.39 mm^2^. (**C**) Quantitative comparison of spatial-temporal resolution trade-offs between 2D global and focal MRI. (**D**) Comparison of full-FOV FSE and rFOV FSE with a fan-shaped phantom. (**E**) Comparison of full-FOV FSE and rFOV FSE with an in vivo human brain imaging. (**F**) Needle tracking results of a simulated brain intervention. (**G**) Tip and angular errors of needle tracking for the simulated human brain intervention. (**H**) Needle tracking for an ex vivo animal tissue intervention. (**I**) Tip and angular errors of needle tracking for an ex vivo animal tissue intervention. The video of (F) and (H) can be found in movie S5.

To address the dual demands of interactive navigation and submillimeter precision in neurointervention, global-focal MRI was developed for robotic intervention (Fig. 4). The sequences implement hybrid imaging protocols during different intervention phases: continuous needle advancement at a constant velocity (e.g., ~0.13 mm/s) with global MRI monitoring (1-mm^2^ in-plane resolution and 2.5-s temporal resolution) when the needle was far from the target (e.g., >10 mm) (Fig. 4A), transitioning to stepwise millimeter-scale movements under high-resolution focal MRI guidance (0.39-mm^2^ in-plane resolution and 17.9-s temporal resolution) when approaching the target (e.g., <10 mm) (Fig. 4B). Here, a 2D gradient echo sequence with golden angle radial sampling (2D GRE radial) was carried out for global MRI with a 1-mm^2^ in-plane resolution and 2.5-s temporal resolution. For volumetric imaging, a 3D gradient echo sequence with stack-of-star (sos) golden angle radial sampling (3D GRE sos radial) was implemented with a 1-mm^3^ isotropic resolution and 40-s temporal resolution. For focal imaging, a 2D reduced field of view fast spin echo (2D rFOV FSE) sequence delivered a 0.39-mm^2^ in-plane resolution and 17.9-s temporal resolution. Although the focal imaging was 7.2-fold slower than 2D global imaging, its 2.6-fold higher spatial resolution proved critical for final phase targeting (Fig. 4C). Phantom validation demonstrated effective suppression of blurring and ringing artifacts around the end of the fan using rFOV FSE compared to conventional full-FOV FSE sequence (Fig. 4D). Compared with conventional imaging with an in-plane resolution of 0.7 mm^2^, rFOV can achieve an in-plane spatial resolution of 0.23 mm^2^. In vivo validation revealed clear visualization of internal cerebral veins and the contour of subthalamic nucleus (STN) using rFOV FSE with the resolution of 0.31 and 0.39 mm^2^ (Fig. 4E).

A Segment Anything Model (SAM)–based needle tracking algorithm provided needle localization from global-focal MRI (fig. S16B), enabling closed-loop robotic control through continuous tip-to-target distance updates. In a simulated human-brain intervention, the algorithm achieved a mean tip position error of 0.95 pixels and a mean angular error of 0.11° (Fig. 4, F and G). Ex vivo validation using porcine brain tissue demonstrated comparable performance with 0.79-pixel position error and 0.69° angular deviation during active insertion (Fig. 4, H and I), confirming clinical-grade tracking precision.

### Phantom, cadaveric, and in vivo animal studies

**Fig. 5.**
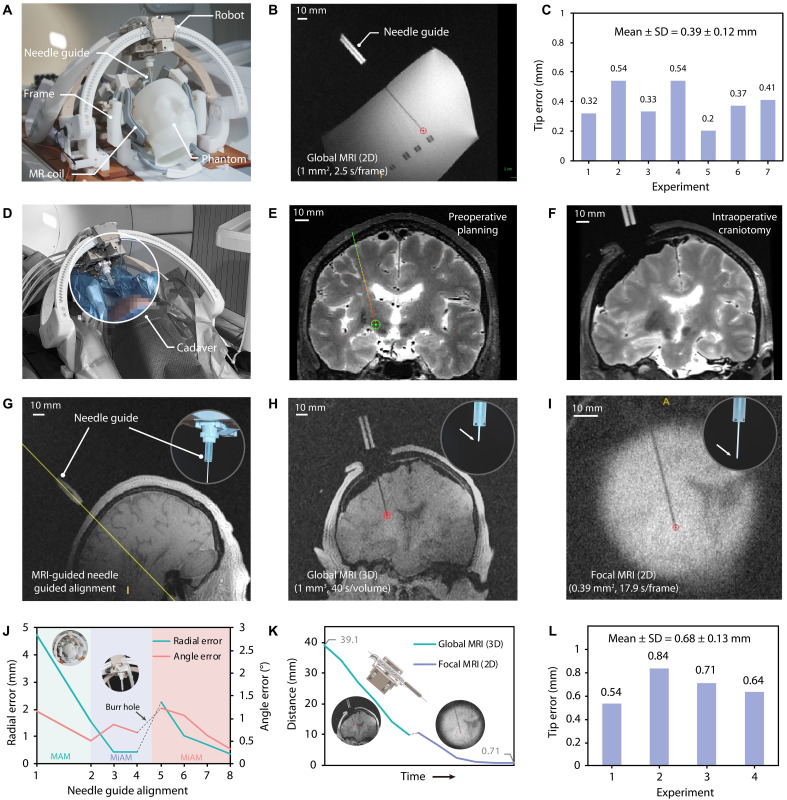
Phantom validation and cadaveric experiments of the MRI-guided robotic system. (**A**) Experiment setup of phantom validation using a 3D-printed skull filled with gelatin containing ten fiducial markers, mounted onto the robot via stereotactic head frame. (**B**) 2D global MRI during the robotic intervention in a phantom experiment with an in-plane resolution of 1 mm^2^ and a temporal resolution of 2.5 s per frame. (**C**) Quantitative analysis of targeting accuracy across seven phantom trials. (**D**) Experimental setup of the cadaver study. (**E**) Preoperative 3D T2-weighted MRI (0.8 mm by 0.8 mm by 0.8 mm) for trajectory planning. (**F**) Intraoperative 3D T2-weighted MRI (0.8 mm by 0.8 mm by 0.8 mm) after craniotomy. (**G**) MRI-guided needle alignment. (**H**) Global MRI during robotic intervention for the cadaver study. (**I**) Focal MRI during the robotic intervention. (**J**) Process of needle alignment in the cadaveric experiment. The robotic system aligned the needle guide with the planned trajectory through task-hierarchical control (Step-2 and Step-3). After the burr hole was created, owing to structural shift, the target and trajectory were updated on the basis of intraoperative MRI. The needle guide was then realigned to the updated path. (**K**) Continuous distance monitoring between the needle tip and target during robotic advancement in the cadaveric experiment. (**L**) Quantitative analysis of targeting accuracy across four cadaveric experiments.

Phantom experiments were conducted to evaluate the accuracy of the system. The experimental setup consisted of a gelatin phantom, an MR head coil, the robot, and a needle guide (Fig. 5A). All insertion procedures used a cone-tipped, MR-safe ceramic needle with an outer diameter of 1.6 mm. The phantom was secured to the robot using a head frame. The entire robot was fixed to the scanner bed. It can move in and out of the MR bore along with the bed. The phantom was prepared by filling gelatin into a 3D-printed human skull mold. Plastic markers were embedded into the gelatin phantom to simulate the targets (Fig. 5B). On the basis of the 3D MRI of the phantom, a plastic marker was chosen as the target, and the interventional path was planned. Then, under the guidance of 3D MRI, the robot aligned the needle guide with the planned trajectory through macro-micro actuation (fig. S17, A and G). Once complete, the needle was manually inserted into the gelatin to a depth of ~10 mm. Then, the soft actuator inserted the needle toward the target under global-focal MRI guidance (Fig. 5B and fig. S17, B, C, and H). Postexperiment 3D MRI scans were performed to quantify the targeting accuracy by measuring the distance between the needle tip and the selected target in three dimensions. In seven independent trials, the tip error was consistently less than 1 mm (mean: 0.39 ± 0.12 mm), demonstrating submillimeter targeting precision of the system (Fig. 5C).

**Fig. 6.**
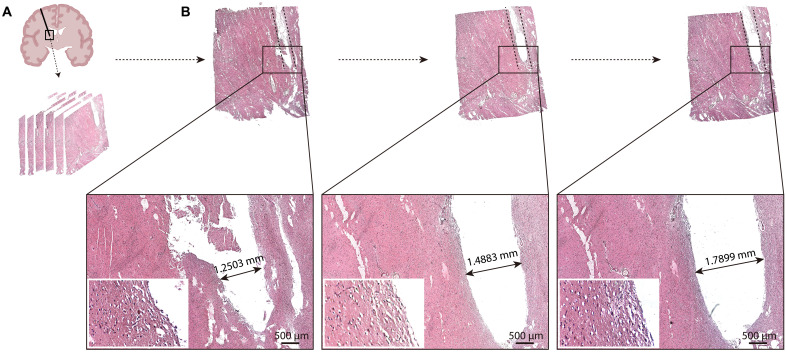
Cadaver brain tissue sections in the STN implantation experiment. (**A**) Schematic diagram of the path and the sampling position. (**B**) Slices of different layers with the needle path and their magnified images. The sidewall of the needle path was magnified, and the width of the needle path was measured at each layer.

To evaluate clinical feasibility, four cadaveric experiments were conducted under neurosurgical operating conditions (Fig. 5D). Preoperative MRI (3D T2-weighted sequence) was obtained for surgery planning (Fig. 5E). The STN was designated as the target, positioned at an average depth of ~65 mm from the cortical surface of the cadaver. Subsequently, the robotic system aligned the needle guide with the planned trajectory (Fig. 5, G and J). Following craniotomy, intraoperative MRI (3D T2-weighted sequence) was performed to assess the structural shift (Fig. 5F). Owing to the notable structural shift, the target and trajectory were updated on the basis of intraoperative MRI. The needle guide was realigned to the updated path using 3D MRI guidance (Fig. 5J). The needle was manually inserted into the brain tissue to a depth of ~10 mm. Then, under global-focal MRI guidance, the robot precisely inserted the ceramic needle to the target (Fig. 5, H, I, and K, and fig. S17, E and F). Postprocedural 3D MRI quantification demonstrated a final targeting accuracy of 0.68 ± 0.13 mm, confirming the system’s ability to achieve submillimeter precision despite intraoperative anatomical changes (Fig. 5L). Histological analysis of a cadaveric specimen (Fig. 6) revealed a maximum needle tract width of 1.79 mm in three slices after insertion using a 1.6-mm needle.

To further demonstrate the performance of the proposed robotic system, an in vivo animal experiment was conducted using a swine. The left nucleus accumbens septi was selected as the target on the basis of the preoperative 3D T2-weighted MRI (fig. S18A). The robotic system then aligned the needle guide with the planned path (fig. S18D). Because no notable shift was observed from intraoperative 3D T2-weighted MRI after craniotomy, the needle guide was not adjusted (fig. S18A). The ceramic needle was manually inserted into the porcine brain to a depth of ~5 mm. Subsequently, stepwise robotic intervention was carried out under global-focal MRI guidance (fig. S18, B, C, and E). The targeting accuracy of the in vivo animal study was 0.14 mm, as determined by the postoperative 3D T2-weighted MRI (fig. S18A). The planned trajectory aimed to minimize iatrogenic damage to the gray matter and surrounding blood vessels. Immediately following needle insertion, the brain tissue was excised and embedded in paraffin. Subsequent pathological sectioning, along with hematoxylin and eosin (H&E) and Nissl staining, revealed that the insertion tract was situated in the white matter region, devoid of neuronal enrichment (fig. S19). The morphology of the neurons appeared normal, indicating that they were not adversely affected by the procedure.

## DISCUSSION

We proposed herein a robotic system for fully-actuated stereotactic positioning and closed-loop intervention with submillimeter accuracies. 3D global MRI was used for stereotactic positioning, whereas 2D global and high-resolution focal MRI guidance was used for needle insertion. In phantom experiments, the system achieved a targeting accuracy of 0.39 ± 0.12 mm (*N* = 7), whereas cadaveric experiments targeting STN showed 0.68 ± 0.13 mm (*N* = 4) accuracy. One in vivo trial showed a 0.14-mm targeting error. Compared to existing commercial manual intraoperative MRI-guided stereotactic frames, our system enables fully-actuated, stable positioning and insertion. Through MRI-based closed-loop feedback, the system provides interactive visualization during intervention, greatly enhancing the safety and precision of the procedures. The system integrates global-focal MRI for navigation and closed-loop control during intervention and precise identification of the approaching target. It also addresses the limitations of commercial stereotactic frames in clinical applications, which require patients to repeatedly move in and out of the MRI scanner to obtain intraoperative adjustment. Furthermore, compared to existing stereotactic robotic systems, this study achieves submillimeter positioning accuracy and realizes fully-actuated large-range positioning and insertion.

The hybrid pneumatic-hydraulic actuation system exhibits excellent MR compatibility. Using pneumatic actuation alone was attempted in several previous studies, and the capabilities such as high load, self-locking, and sensor-free step control were appreciated (*17*, *21*, *33*, *34*). Our previous research confirmed that this pneumatic actuation could achieve a stable performance during MRI guidance (*34*). In this work, the step pneumatic actuators have good structural compatibility, enabling a simple mechanism to achieve large-range motion without relying on position sensors, demonstrating broad application potential in MRI environments. Although being fast, the pneumatic actuator has a relatively large error dictated by the step size. To address this limitation, precise hydraulic micropositioning is deployed, and resulting in the hybrid macro-micro actuation scheme as proposed in this paper. The hydraulic actuator exhibits outstanding repeatability and reliable accuracy. The proposed hybrid macro-micro pneumatic-hydraulic actuation not only has excellent MR compatibility and minimal SNR_loss_ and distortion but also resolves the trade-off in achieving concomitant large-range operation and high-precision positioning in confined neurosurgical environments. The 4-df macroconfiguration enables large-range stereotactic brain positioning, required for procedures such as bilateral brain implantation in DBS procedures. The system demonstrates high stiffness (Fig. 2E, *x*: 12.64 N/mm, *y*: 12.04 N/mm, and *z*: 35.96 N/mm), a critical feature for high-precision neurosurgical procedures. The hydraulic actuator also has the potential for further miniaturization, making it suitable for multi-df microoperations and cost-effective disposable designs. This advancement paves the way for single-use disposable robotic systems in MRI-guided intervention.

The bioinspired soft actuator for needle insertion resolves key limitations of structural complexity, bulkiness, and poor MR compatibility in decoupled automatic control mechanisms for needle rotation and insertion. Fabricated via 3D soft-printing with MR-safe photocurable polymers, the soft actuator uses a helical locking mechanism with strong locking strength (13 N; Fig. 3K) and a puncture force of up to 1.7 N during insertion (Fig. 3J). This actuator reliably supports surgeons in performing biopsy, ablation, and implantation. Its performance demonstrates excellent motion stability for brain insertion under MRI navigation (movies S6 and S7). In addition, this peristaltic delivery method enables theoretically unlimited insertion depths through the continuous propulsion design. This fabrication strategy allows customizable rotation/delivery speed ratios through structural parameter optimization for specific procedures. For scenarios requiring higher puncture forces, for example, increasing the translation ratio can amplify delivery force output. Traditional actuation designs (e.g., piston or lead-screw systems) inherently suffer from excessive bulk when requiring combined rotary and linear motion. By contrast, this compact (Φ18.4 mm, 30 g), easy-to-manufacture (3D printing), disposable, stable (locking force: 13 N), and cost-effective (~$5) needle insertion actuator holds substantial potential for widespread application in future clinical scenarios.

The proposed global-focal MRI framework provides an effective strategy for balancing temporal resolution and spatial precision in intraoperative MRI. By combining two complementary scanning modes—continuous low-resolution tracking during rapid needle advancement (0.13 mm/s) and high-resolution stepwise verification near targets—the system achieves superior spatial accuracy (0.39-mm^2^ in-plane resolution) while maintaining interactive monitoring capabilities. Beyond spatial resolution enhancements, the integration of SAM-based needle tracking with rFOV FSE sequences enables interactive tip localization. Phantom and cadaveric experiments confirmed a positional accuracy of 0.79 pixels, stemming from the synergy between rFOV FSE’s high spatial resolution and SAM’s segmentation precision. Notably, SAM’s zero-shot generalization capability ensured robust needle identification against heterogeneous brain tissue environments without requiring extensive annotated datasets—a notable advantage over prior deep learning approaches (*35*) that typically demand large annotated datasets, which are particularly challenging to obtain in neurosurgical settings. The system simultaneously visualizes the instrument and surrounding tissues with high spatiotemporal resolution. This capability facilitates interactive procedural guidance through live imaging feedback, addressing a critical unmet need in neurological interventions. Cadaveric experiments validated the system’s ability to compensate for intraoperative brain shift, a persistent limitation of conventional frame-based methods. The combined technical advancements highlight the platform’s potential to improve precision and reliability in clinical practice. Although the current focal MRI acquisition time (17.9 s) aligns with clinical safety margins, further acceleration through compressed sensing or deep learning could optimize workflow efficiency. The current robotic positioning duration (30 min) can be optimized via precise calibration of kinematic parameters and enhanced control algorithms, potentially decreasing iterations from four-to-seven cycles to one or two cycles. Concurrently, optimizing MRI scanning protocols to ≤1 min per sequence would collectively enable accurate targeting within 10 min, matching the efficiency benchmark of non–MR-compatible robotic systems [10 min (*36*)].

Performing fully-actuated robotic positioning within MRI environments carries recognized clinical benefits: Intraoperative MRI necessitates operating within the confined MRI bore where manual manipulation proves exceedingly challenging. Moreover, manual operation relies on surgeon expertise, escalating institutional training costs. Fully-actuated systems not only shorten the learning curve but also enable remote operation—deployable in remote regions requiring only on-site assistants for basic setup and instrument changes, whereas experienced surgeons guide or teleoperate the entire procedure remotely. In the future, human-robot collaborative frameworks may better align with clinical workflows. Surgeons would retain overriding authority for critical decisions and intraoperative verification—for instance, confirming target alignment via intraoperative MRI before instrument advancement, rather than relying solely on positioning accuracy. Integrating force-sensing needles would allow surgeons to telemanipulate insertion via handheld controllers, with immediate halt protocols triggered by abnormal resistance (e.g., vascular encounters). Such assisted decision-making, positioning, and insertion methodologies would likely accelerate clinical adoption.

Although our system achieved the submillimeter targeting accuracy in vivo/vitro, outperforming non–MR-compatible neurosurgical robots [~1.5 mm (*36*)], its positioning repeatability (0.18 mm) was lower than conventional serial manipulators [typically 0.1 mm (*36*)]. To address this limitation, future precision enhancements may be realized through optimization of the manufacturing process from laboratory settings to industrial scale development, as well as the development of high-precision MR-compatible sensors. Although stereotaxy provides foundational targeting, its rigid instrumentation cannot compensate for intraoperative tissue shifts, limiting deep tissue accuracy. The development of MR-compatible steerable needles (*37*, *38*) could substantially enhance clinical applicability. However, such steerable needles must simultaneously deliver three critical capabilities: dynamic nonlinear trajectory control, closed-loop path tracking, and avoidance of tissue laceration. Techniques such as the MR field-actuated flexible needle or thin continuum robot systems with path-following capabilities would offer future solutions for curved deep brain interventions (*38*).

This study addresses the lack of intraoperative MRI guidance and inherent limitations in manual neurosurgical procedures. The proposed system has scope for further development. In the design of pneumatic and hydraulic actuators, the high-precision 3D printing technology enables more miniaturized configuration for more portable clinical applications. Considering the difficulty of obtaining accurate theoretical models for the complex chambers of the bioinspired actuator, combining machine learning (*39*) with FEA models could optimize parameters for diverse clinical needs. Multimodal intraoperative MRI is crucial for intervention, and richer modalities beyond basic tissue structures can provide surgeons with more comprehensive information. Furthermore, integrating force sensing (*40*, *41*) into the needle can further improve procedural safety and consistency. Although the clinical adoption of intraoperative MRI suites is thus far limited, with continuing technological advancement and price reduction, coupled with maturity of mobile low-field MRI units (*42*), broader adoption by medical institutions is to be expected. These would ultimately enable safer and more effective robotic-assisted neurosurgical interventions.

## MATERIALS AND METHODS

### Pneumatic step actuator

The pneumatic step actuator was driven by two pistons (fig. S3), each controlled by a 5/2-way solenoid valve (MHP2-MS1H-5/2-M5, Festo), which alternately pressurized one piston chamber with compressed air and exhausted the opposing chamber to atmosphere. During actuation, pressurization of one piston chamber forced engagement with the output gear. Bidirectional motion was achieved by alternating the pressurization sequence of the two pistons (fig. S3A). Consequently, this stepping pneumatic actuator enables discrete positioning close to the predefined targets, obviating the need of closed-loop end-effector sensing. In the rotational actuator configuration, the two pistons were orthogonally arranged [Fig. 2A, (d)], whereas for the arc motion [Fig. 2A, (a)] and rotation on the arc [Fig. 2A, (b)], the two pistons were distributed along the output gear. Stepping motion was on the basis of the engagement of one piston with the output gear at the θ/4 position, whereas the other piston was fully engaged (fig. S3B). The step of the rotational pneumatic motor was 10° per step, whereas the arc motion and rotation on the arc were both 0.5° per step. A controller (MEGA2560, Arduino) outputted a 5- and 0-V pulse width modulation (PWM) signal to control the switching of each solenoid valve via a 24-V MOSFET board (fig. S3C). The shielded control console was connected to the pneumatic step actuator via 3-m-long pneumatic tubes for remote operation.

### Macropneumatic stereotactic actuation

The first df translation and second df lifting motion were driven by the rotational pneumatic motor with the polyether ether ketone (PEEK) lead screw (4-mm pitch), achieving high-resolution translation (4/36 = 0.11 mm per step) and stable lifting (≈0.1° per step). To minimize the size of the actuation module, a needle roller bearing was used for translational guidance, and these two lead screws were paralleled, resulting in an overall height of 48.5 mm for the first df and second df actuation. For the third df actuation [Fig. 2A, (a)], the output gear was integrated into the arc, with the actuation pistons distributed on both sides of the arc. This configuration enabled controlled movement along the 200-mm-radius arc trajectory. Similarly, a smaller output gear was mounted on the arc to achieve rotation [fourth df; Fig. 2A, (b)] around the *x* axis with the motion radius 70 mm. The arc motion featured an RCM (remote center of motion) point at the center of the system because of the combination of the second df and third df. The RCM point coincided with the center of the MRI bore, ensuring that the brain was positioned as close to the MRI center as possible for the best imaging quality.

### Master-slave hydraulic actuator

The master piston was housed within the control console, with the hydraulic fluid transmitted through the 3-m-long hydraulic pipeline to the slave piston (fig. S4A). By applying a prepressurization system that maintains 0.1 to 0.3 MPa prepressure, the hydraulic system can directly bypass the low-pressure filling phase during start-up (fig. S4B). On the basis of the incompressibility of the hydraulic fluid, the motor drove the master piston to push the fluid to the slave piston, thereby achieving synchronized motion between the master and the slave. The cross-sectional area of the piston cylinder on the A side at both the master and slave was 113 mm^2^, whereas the B side was 85 mm^2^. The elasticity of the hydraulic pipelines and piston friction caused pressure fluctuations during reversal. These factors led to changes in fluid volume and results in hysteresis effects. Hysteresis can be effectively controlled by exploiting the pressure-switching repeatability of master-slave hydraulic pistons during unidirectional motion, thereby enabling absolute position control of the actuator (fig. S20). The master piston was actuated by an electric motor (Dynamixel MX-106, ROBOTIS), designed to achieve precise motion with a 4-mm pitch lead screw. Dimethyl silicone oil (20 centistokes) was used as the filling fluid to ensure lubricating properties and to reduce hydraulic resistance in the pipeline. Before system activation, a prepressurization step (0.3 MPa) was applied to both pipelines to eliminate microbubbles and provide preexpansion of the pipelines, thereby mitigating the hysteresis effect.

### Microhydraulic actuation

The hydraulic actuators were integrated into the 4-df MiAM system (fig. S5A and movie S2) in a parallel configuration on the basis of linear motion. The hydraulic actuators were arranged perpendicularly to each other in each plane, allowing for 4-df fine adjustment. Each hydraulic actuator was connected to a rod perpendicular to its position in the *x*-*y* plane, linked by L-shaped sliders to two rods with a height difference. The connections were equipped with brass bushings to ensure lubrication and coaxiality. Two linear active rods and two passive rods formed the cubic workspace. During the swinging motion adjustment, the length of the link between the two planes varied. Therefore, universal joints were designed for linkage, with the upper part fixed to the link and the lower part connected to the link via a cylindrical bushing to enable relative sliding (fig. S5B). Regarding the fine adjustment, the main sources of error in the MAM included insufficient engagement between the active and passive gears during stepping motion, mechanical assembly errors, and joint size calibration errors. The cumulative error was limited to no more than one pneumatic step length combined with mechanical assembly errors and joint size calibration errors. By simulating one step of each joint in the macromotion as the maximum error based on the macro-micro kinematic model, the maximum joint movement range for microactuation was determined to be ±6.5 mm.

### Geometric modeling of the bioinspired soft actuator

As shown in fig. S8, axially twisting the set of linear channels circumferentially arranged around the needle enables the conversion of rotational motion into helical conveying motion along an inclined path. The geometric models of fig. S8 (B to D) were constructed using the equations of cylindrical helices as follows

$$\left\{ \begin{array}{c} x\left(\theta_{\xi }\right)=R_{\xi }\text{cos}\left(\chi_{\xi }\theta_{\xi }+\Delta \theta_{\xi }\right)\\ y\left(\theta_{\xi }\right)=R_{\xi }\text{sin}\left(\chi_{\xi }\theta_{\xi }+\Delta \theta_{\xi }\right)\\ \;z\left(\theta_{\xi }\right)=R_{\xi }\theta_{\xi }\text{tan}\left(\alpha_{\xi }\right) \end{array}\right.$$ (1)

where $R_{\xi }$ denotes the primary helical radius, $\theta_{\xi }$ represents the helix angle, $\chi_{\xi }=1,-1$ indicates the helix direction, $\Delta \theta_{\xi }$ signifies the initial phase angle of the helix, and $\alpha_{\xi }$ represents the lead angle of the helix. When the helix angle ( $\alpha_{\xi }$ ) approaches 0°, the rotational component predominates in the helical motion. Conversely, when the helix angle ( $\alpha_{\xi }$ ) approaches 90°, the translational component predominates in the helical motion. The pitch $h_{\xi }$ of the helical structure is governed by the following equation

$$h_{\xi }=2\pi R_{\xi }\text{tan}\left(\alpha_{\xi }\right)$$ (2)

By introducing angular undulation components in *X* and *Y* directions, spatial decoupling of intersection points could be achieved (fig. S9E). The primary helix evolved into a secondary helix (*43*) under angular undulation, with its equation as follows

$$\begin{array}{c} \left\{ \begin{array}{c} x\left(\theta_{\xi }\right)=\left[R_{\xi }+\chi_{\xi }r_{\xi }\text{cos}\left(\theta_{\xi }n\right)\right]\text{cos}\left(\chi_{\xi }\theta_{\xi }+\Delta \theta_{\xi }\right)\\ y\left(\theta_{\xi }\right)=\left[R_{\xi }+\chi_{\xi }r_{\xi }\text{cos}\left(\theta_{\xi }n\right)\right]\text{sin}\left(\chi_{\xi }\theta_{\xi }+\Delta \theta_{\xi }\right)\\ \;z\left(\theta_{\xi }\right)=R_{\xi }\theta_{\xi }\text{tan}\left(\alpha_{\xi }\right) \end{array}\right. \end{array}$$ (3)

where $r_{\xi }$ represents the amplitude of the second-order helix. *n* represents the number of secondary helices per primary helix cycle, equivalent to the number of intersections between the CW and CCW chambers.

### FEA of the bioinspired soft actuator

To verify the design, we established an FEA model in Abaqus for the bioinspired soft actuator (Fig. 3, F and G, figs. S10 to S12, and movie S3). The boundary of the soft actuator was constrained in *x*, *y*, and *z* directions to simulate its installation on the MiAM system. Contact interaction was defined between the needle surface and the actuation surface of the soft actuator to characterize the variation in contact stress induced by the traveling wave motion. The soft actuator was modeled as a hyperelastic material with the strain energy potential defined by the Yeoh model. The hyperelastic coefficients were specified using as *C*_10_ = 0.11, *C*_20_ = 0.02, with a third-order strain energy potential. The needle material was characterized by a Young’s modulus of 210 GPa and a Poisson’s ratio of 0.3. The tangential friction behavior was modeled using the Penalty friction formulation with a friction coefficient of 0.3. The normal response was specified with a pressure-overclosure setting of linear and a contact stiffness of 0.3.

### Kinematics and control of the macroactuation system

The homogeneous transformation matrix **T** of the robot was used to establish the macroforward kinematics model on the basis of the Denavit-Hartenberg (D-H) method (*44*) as follows

$$\begin{array}{c} T=\left[\begin{array}{ccc} \;c\alpha & s\alpha c\beta s\gamma +s\alpha s\beta c\gamma & \;s\alpha c\beta c\gamma -s\alpha s\beta s\gamma \;d+Ds\alpha c\beta \\ 0 & c\beta c\gamma -s\beta s\gamma & \begin{array}{cc} \;-c\beta s\gamma -s\beta c\gamma & \;-Ds\beta \end{array}\\ \begin{array}{c} -s\alpha \\ 0 \end{array} & \begin{array}{c} c\alpha c\beta s\gamma +c\alpha c\gamma s\beta \\ 0 \end{array} & \begin{array}{cc} \begin{array}{c} c\alpha c\beta c\gamma -c\alpha s\beta s\gamma \\ 0 \end{array} & \begin{array}{c} Dc\alpha c\beta \\ 1 \end{array} \end{array} \end{array}\right] \end{array}$$ (4)

where *c* and *s* represent cos (•) and sin (•), respectively. The equation included the four macrojoint parameters *d*, α, β, and γ, which have already been defined, and the arc radius *D*. The end-effector of this kinematic model coincided with the base frame of the MiAM. The Jacobian matrix **J** of MAM was computed by differentiating **T** with respect to the joint variables $q_{\text{mac}}={\left[d,\alpha ,\beta ,\gamma \right]}^{T}$ . An iterative inverse kinematics solver was formulated using the pseudoinverse method

$$q_{\text{mac},k+1}=q_{\text{mac},k}+\eta J^{†}\left(q_{\text{mac},k}\right)\Delta P_{k}$$ (5)

where $\Delta P_{k}$ represents the pose error (3D positional and orientation offsets) of the end-effector. $q_{\text{mic},k}$ represents the macrojoint parameters at the *k*th iteration. $J^{†}={\left(J^{T}J+\lambda_{m}I\right)}^{-1}J^{T}$ is the damped pseudoinverse, ensuring that $J^{T}J+\lambda_{m}I$ remains invertible when approaching singularity. It balances error minimization with joint velocity norms to prevent excessive joint velocities. η represents the step size scaling factor to prevent overshooting. Equation 5 enabled both inverse kinematics resolution and error compensation.

### Kinematics and control of the microactuation system

The microactuator was designed to move along the *x* axis and *y* axis. Thus, the forward kinematics could be directly derived from the coordinate systems. Using the trajectory projection method, the offsets were mapped onto the coordinate systems of the upper and lower planes. In addition, because the end-effector of the MAM serves as the base frame for the MiAM, the iterative inverse kinematics solver could be derived as follows

$$q_{\text{mic},i+1}=q_{\text{mic},i}+{\mathrm{Kf}}_{\text{mapping}}\left(\Delta P_{i}\right)$$ (6)

where $q_{\text{mic},i}$ represents the macrojoint parameters at the *i*th iteration, $f_{\text{mapping}}\left(x\right)$ represents the projection of the trajectory $\Delta P_{k}$ onto the upper and lower planes. *K* represents the step size scaling factor to prevent overshooting. By calculating the projection between the end-effector (with orientation tracked via 3D MRI) and the planned trajectory and then iteratively applying Eq. 6, the planned trajectory was asymptotically approximated.

### Task hierarchical control strategy

The task prioritization strategy was used to analyze the input data and determine the actions of the robot (fig. S7). The execution procedure consisted of four steps: Step 1: The robot performed initial macromotions toward the planned target using macroinverse kinematics. Step 2: Macroadjustment. 3D MRI guidance directed the MAM to move incrementally through the macroiterative algorithm until the MRI navigation indicated that the positional error was less than 2 mm and the angular error was below 1°. Step 3: Microadjustment. This stage used the latest **T** matrix of MAM to determine the position of the base of the MiAM. Similarly, 3D MRI guidance directed the MiAM to make the required fine adjustment. The process iterated through microinverse kinematics until the robot achieved the required accuracy (position error < 0.5 mm; angular error  < 0.3°). Step 4: Needle insertion. The needle mounted on the bioinspired soft actuator was advanced while the MRI switches between the global mode and local mode for interactive monitoring of the trajectory of the instrument. The trajectory and positional deviation between the needle tip and the target were continuously computed. The soft actuator maintained needle positioning until the positional deviation fell below 0.5 mm during closed-loop control.

### MR compatibility

The MR compatibility of the robotic system was assessed on a 3-T MRI system (uMR790, United Imaging, China). We evaluated both the background noise impact to the signal (SNR_loss_) and geometric distortion caused by the robotic system (Fig. 2B). The SNR_loss_ was defined as the interference of SNR observed in images acquired after introducing the robotic system. The SNR_loss_ measurements were performed using two standard imaging sequences: T1-weighted and T2-weighted. The distortion interference was tested in T2-weighted sequence. The sequence parameters were as follows. T1-weighted for the SNR test: FOV = 256 mm by 256 mm, acquisition matrix = 256 × 256, slice thickness = 5 mm, repetition time (TR)/echo time (TE) = 800/12.32 ms, and bandwidth = 130 Hz/pixel. T2-weighted for the SNR test: FOV = 256 mm by 256 mm, acquisition matrix = 256 × 256, slice thickness = 5 mm, TR/TE = 4000/73.92 ms, and bandwidth = 130 Hz/pixel. T2-weighted for distortion test: FOV = 384 mm by 334 mm, acquisition matrix = 256 × 233, slice thickness = 6.5 mm, TR/TE = 4500/100.8 ms, and bandwidth = 200 Hz/pixel. Quantitative comparisons were performed between the control condition and three different robot states. Baseline MR images were first acquired using the standard phantom: a spherical phantom (Model 8004317, UIH) for SNR evaluation and an ACR MRI accreditation phantom (ACR-PH-1, Gammex) for geometric distortion assessment, with the robotic system absent from the scanner bore. Subsequent acquisitions were performed under three conditions: the robot positioned adjacent to the phantom without power supply (static), the powered robot maintaining stationary position (powered), and the robot executing programmed movements (moving). These MR images were compared against the baseline acquisitions (Fig. 2B and fig. S6).

### MRI navigation

Three different cylindrical MRI navigation markers (needle guide) were developed for needle guidance, featuring a straight PEEK tube serving as the fluid-sealed intermediate channel (fig. S21). The needle guide was fabricated using 3D printing technology. The solution (NiSO_4_·6H_2_O:NaCl:H_2_O = 1.243 g:2.5 g:1000 g) used for intracavitary filling that exhibit an active signal on MRI. The needle guide was rigidly mounted on the MiAM, with its central axis precisely aligned to the bioinspired soft actuator. Following each positional adjustment of the needle guide, 3D MRI was performed to localize the pose of the needle guide. The segmentation was initially performed on the MR images using a threshold-based region growing algorithm. A central axis was subsequently reconstructed on the basis of the binary images. The robotic controller received the 3D spatial vector coordinates of the reconstructed central axis to make the adjustment. The minimum Euclidean distance projection point between the target location and central axis was determined and denoted as p . The Euclidean distance between the point p to the target was defined as the predicted interventional radial error. When the predicted radial error was less than 0.5 mm, the system was considered adequately aligned with the preplanned trajectory, triggering the execution of the robotic-assisted intervention.

### Global MRI

In the phantom study, 2D global MRI acquisitions were performed using a 2D GRE sequence with golden angle radial sampling. Key sequence parameters included the following: FOV = 256 mm by 256 mm, acquisition matrix = 256 × 256, slice thickness = 5 mm, channels = 12, TR/TE = 5.20/2.29 ms, and flip angle = 30°. The 2D global MRI has an in-plane resolution of 1.0 mm by 1.0 mm and a temporal resolution of 2.5 s per frame. For 3D global MRI in the cadaveric study, a 3D GRE sequence with sos golden angle radial sampling was used with the following sequence parameters: FOV = 256 mm by 256 mm by 18 mm, acquisition matrix = 256 × 256 × 18, slice thickness = 1 mm, channels = 12, TR/TE = 5.30/2.43 ms, and flip angle = 10°. The 3D global MRI has an isotropic resolution of 1.0 mm by 1.0 mm by 1.0 mm and a temporal resolution of 40 s per volume.

### Focal MRI

In MRI, the combination of a 1D rf pulse with a 1D gradient can excite a specific 2D plane. This is commonly used for slice selection. Similarly, the combination of a 2D rf pulse with a 2D gradient can excite a 3D cylindrical region. By combining a 2D-selective excitation pulse with a 1D slice-selection pulse, fast high-resolution 2D imaging of a region of interest (ROI) can be achieved. This technique is known as rFOV imaging. In rFOV imaging, wrap-around artifacts are avoided because the region outside the ROI is not excited, allowing *k*-space sampling with larger strides. In this study, for 2D focal MRI, a 2D rFOV FSE sequence was implemented (fig. S16A). The sequence parameters were as follows: FOV = 100 mm by 100 mm, acquisition matrix = 256 × 256, slice thickness = 5 mm, and TR/TE = 1000/75 ms. The 2D focal MRI has an in-plane resolution of 0.39 mm^2^ and a temporal resolution of 17.9 s per frame. The small-tip-angle approximation was used to solve the Bloch equation. The rf pulse waveform *B*_1_(*t*) was defined as follows (*45*)

$$B_{1}\left(t\right)=W\left[k\left(t\right)\right]|ϒG(t)|$$ (7)

$$W\left[k\left(t\right)\right]=\rho e^{-\tau^{2}\left(k_{x}^{2}+k_{y}^{2}\right)/A^{2}}$$ (8)

where *W*[*k*(*t*)] represents the *k*-space weighting function using a symmetric Gaussian function, *G*(*t*) represents the gradient waveform, ϒ represents the gyromagnetic ratio, and *k_x_*, *k_y_* are the *k*-space coordinates with a spiral trajectory. The quantity ρ modulates the flip angle, τ sets the spatial resolution, and the factor *A* governs the size of the spiral trajectory. A 90° flip angle was used for the rf excitation pulse. FSE sequence was a fast imaging sequence that uses an rf excitation pulse followed by several refocusing pulses in an echo train to produce multiple spin echoes (*46*). Here, we incorporated the 2D-selective excitation pulse with an FSE sequence, terming this hybrid approach rFOV FSE. We chose the echo train length of seven to achieve a trade-off between acquisition efficiency and image fidelity.

### Interactive needle tracking

The interactive needle tracking comprised segmentation and localization modules (fig. S16B). In the needle segmentation module, initially, the ROI was manually given [or automatically recognizes using YOLOv8 (*47*)], followed by precise boundary delineation using the SAM (*48*) with the bounding box prompt. In the needle localization module, the needle’s 3D orientation vector and tip coordinates were identified using a least-squares fitting method. Specifically, the needle’s central axis was first extracted from its binary mask, and its parametric equation was determined through a least-squares fitting algorithm. The needle’s endpoints were then pinpointed at the intersections of this line with the mask. The definitive tip position was deduced from these endpoints, considering the needle’s insertion trajectory.

### Phantom study

In the phantom study, all 3D MRI was performed with a 3D GRE sos radial sequence. The 3D MRI for preoperative planning was acquired with the following parameters: FOV = 256 mm by 256 mm by 120 mm, acquisition matrix = 256 × 256 × 120, slice thickness = 1 mm, channels = 12, TR/TE = 5.30/2.43 ms, and flip angle = 10°. The scanning time was 4 min 18 s. The 3D MRI for needle guide alignment was acquired with a volume size of 256 × 256 × 30 and a scanning time of 1 min 06 s. The 3D MRI for accuracy assessment was acquired with the following parameters: FOV = 256 mm by 256 mm by 64 mm, acquisition matrix = 320 × 320 × 80, slice thickness = 0.8 mm, channels = 12, TR/TE = 5.57/2.52 ms, flip angle = 10°, and averages = 2. The resolution was 0.8 mm by 0.8 mm by 0.8 mm, and the scanning time was 6 min.

### Cadaveric study

In the cadaveric study (ethics approval no. 2022329, Institutional Review Board of Ruijin Hospital, Shanghai, China), the preoperative MRI for planning and intraoperative MRI after craniotomy was acquired with a 3D T2-weighted GRE sequence. The sequence parameters included the following: FOV = 240 mm by 256 mm by 166.4 mm, matrix size = 300 × 320 × 208, TR/TE = 3000/456.52 ms, and slice thickness = 0.8 mm. These parameters resulted in a voxel volume of 0.8 mm by 0.8 mm by 0.8 mm. The scanning time was 4 min 42 s. The 3D MRI for needle guide alignment was acquired with a volume size of 256 × 256 × 40 and a scanning time of 1 min 27 s. The parameters of 3D MRI for accuracy assessment were the same as the phantom study.

### In vivo animal study

A swine (3 months old, 30 kg, ethics approval no. SHYS-SOP1-031-F003 A/0, Silver Snake Clinical Center, Shanghai, China) was used for the in vivo animal study (fig. S22). Preoperative MRI for planning, intraoperative MRI after craniotomy, and postoperative MRI for accuracy evaluation were all acquired with a 3D T2-weighted GRE sequence. The sequence parameters were as follows: FOV = 240 mm by 256 mm by 166.4 mm, matrix size = 300 × 320 × 208, TR/TE = 3000/456.52 ms, and slice thickness = 0.8 mm. These parameters resulted in a voxel volume of 0.8 mm by 0.8 mm by 0.8 mm. The scanning time was 4 min 42 s. Preoperative procedures, including coordinate system registration, MRI scan, and surgical planning, took ~36 min. The intraoperative phase, from robotic needle guide alignment to the robot completing the intervention, lasted about 91 min. The robotic localization and 3D MRI navigation during this procedure requires 30 min. The postoperative MRI scan and accuracy evaluation took ~20 min.

### Pathological section study

For H&E staining (Fig. 6, A and B, and fig. S19, A and B), tissue sections were deparaffinized, rehydrated, and stained with hematoxylin (Beyotime, #C0105S) for 5 min, followed by rinsing in running water. Subsequently, eosin (Beyotime, #C0105S) was applied for 5 min. After costaining, the sections were dehydrated and mounted with neutral resin. Images were captured using the bright-field view of a fluorescence microscope. For Nissl staining (fig. S19, C and D), sections were stained with methylene blue dye for 10 min and then rinsed with distilled water. Differentiation was performed using the Nissl staining solution. Last, the sections were dehydrated with graded ethanol, cleared with xylene, and mounted with neutral resin.
