# Supplementary material for: Hybrid pneumatic-hydraulic actuation for MRI-guided robotic stereotactic neurointervention
Source: Sci Adv. 2025 Sep 3;11(36):eady3624. doi: 10.1126/sciadv.ady3624 (PMC12407083; doi:10.1126/sciadv.ady3624)
Supplement: Supplementary file 1 — Figs. S1 to S22 Table S1 Legends for movies S1 to S7 [file sciadv.ady3624_sm.pdf]

Science Advances
AAAS

Supplementary Materials for

Hybrid pneumatic-hydraulic actuation for MRI-guided robotic stereotactic neurointervention

Shaoping Huang *et al.*

Corresponding author: Qingfang Sun, rjns123@163.com; Yuan Feng, fengyuan@sjtu.edu.cn; Anzhu Gao, anzhu_gao@sjtu.edu.cn; Guang-Zhong Yang, gzyang@sjtu.edu.cn

Sci. Adv. **11**, eady3624 (2025)
DOI: 10.1126/sciadv.ady3624

The PDF file includes:

Figs. S1 to S22
Table S1
Legends for movies S1 to S7

Other Supplementary Material for this manuscript includes the following:

Movies S1 to S7

Supplementary Figures

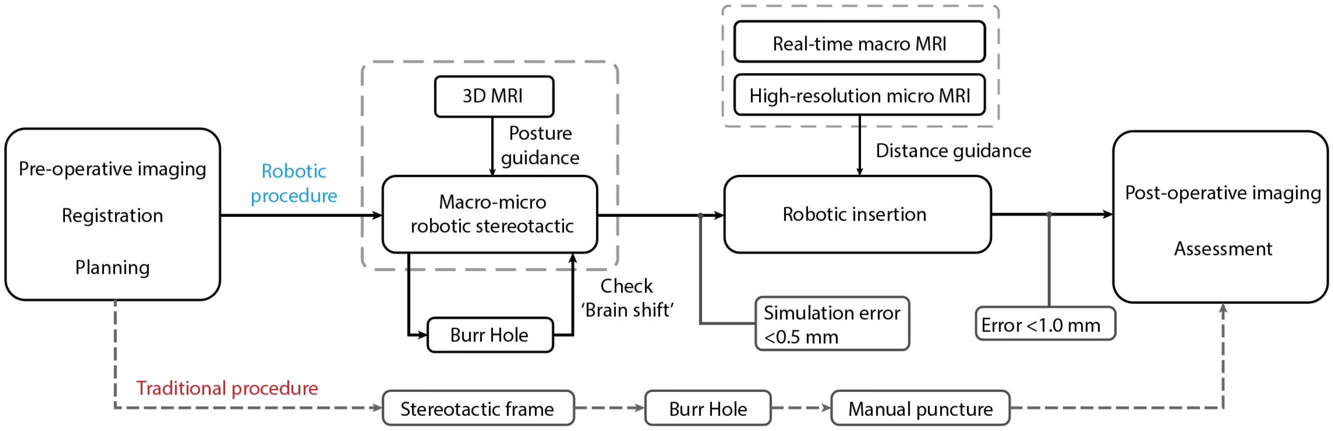

Supplementary Figure 1. Robotic neurointerventional surgical procedure proposed in this paper.

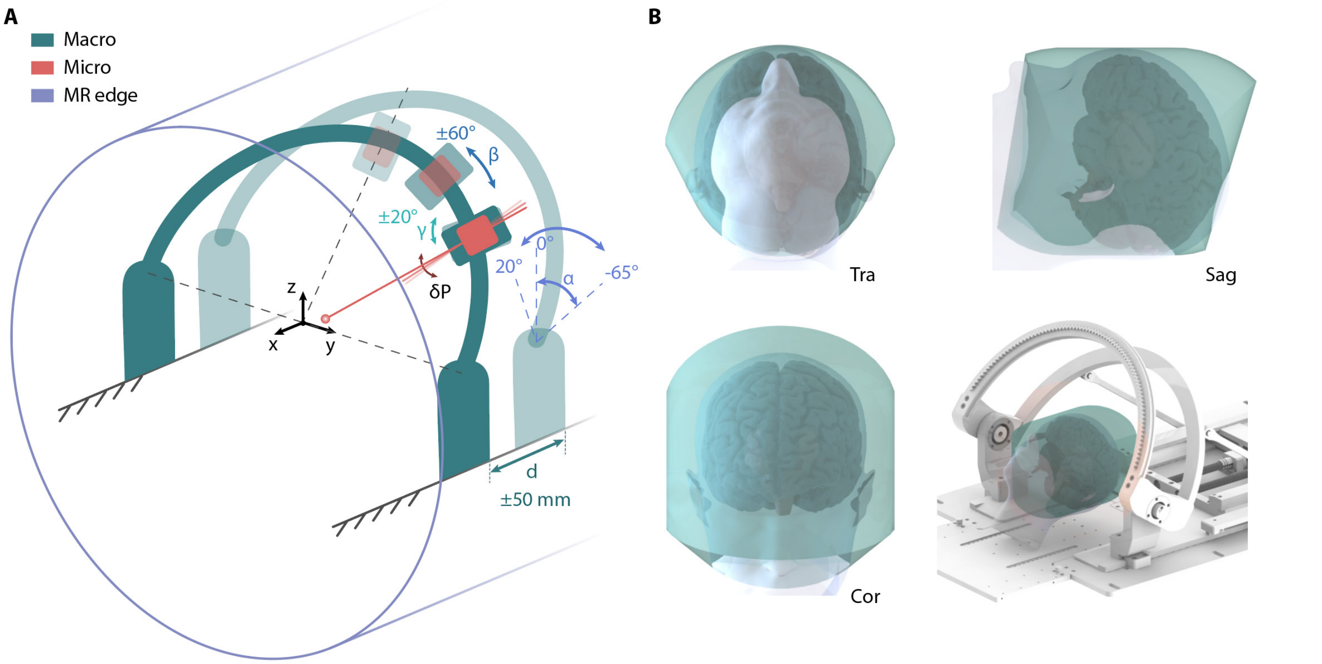

Supplementary Figure 2. Macro-micro adjustment and macro workspace analysis. (**A**) Macro-actuation DoF. Macro actuation provides four DoF: translational displacement d (1st DoF), lifting motion α (2nd DoF), arc motion β (3rd DoF) and rotation on the arc γ (4th DoF). The pink component represents the MiAM integrated with the MAM. When the MAM approaches the planned trajectory, the MiAM executes fine adjustments δP to achieve submillimeter trajectory alignment. (**B**) Macro-actuation workspace derived from forward kinematics. A human brain model is depicted with uniform dimensions, and the green region represents the macro workspace. The workspace is visualized from three planes views: sagittal (Sag), transverse (Tra), and coronal (Cor) planes.

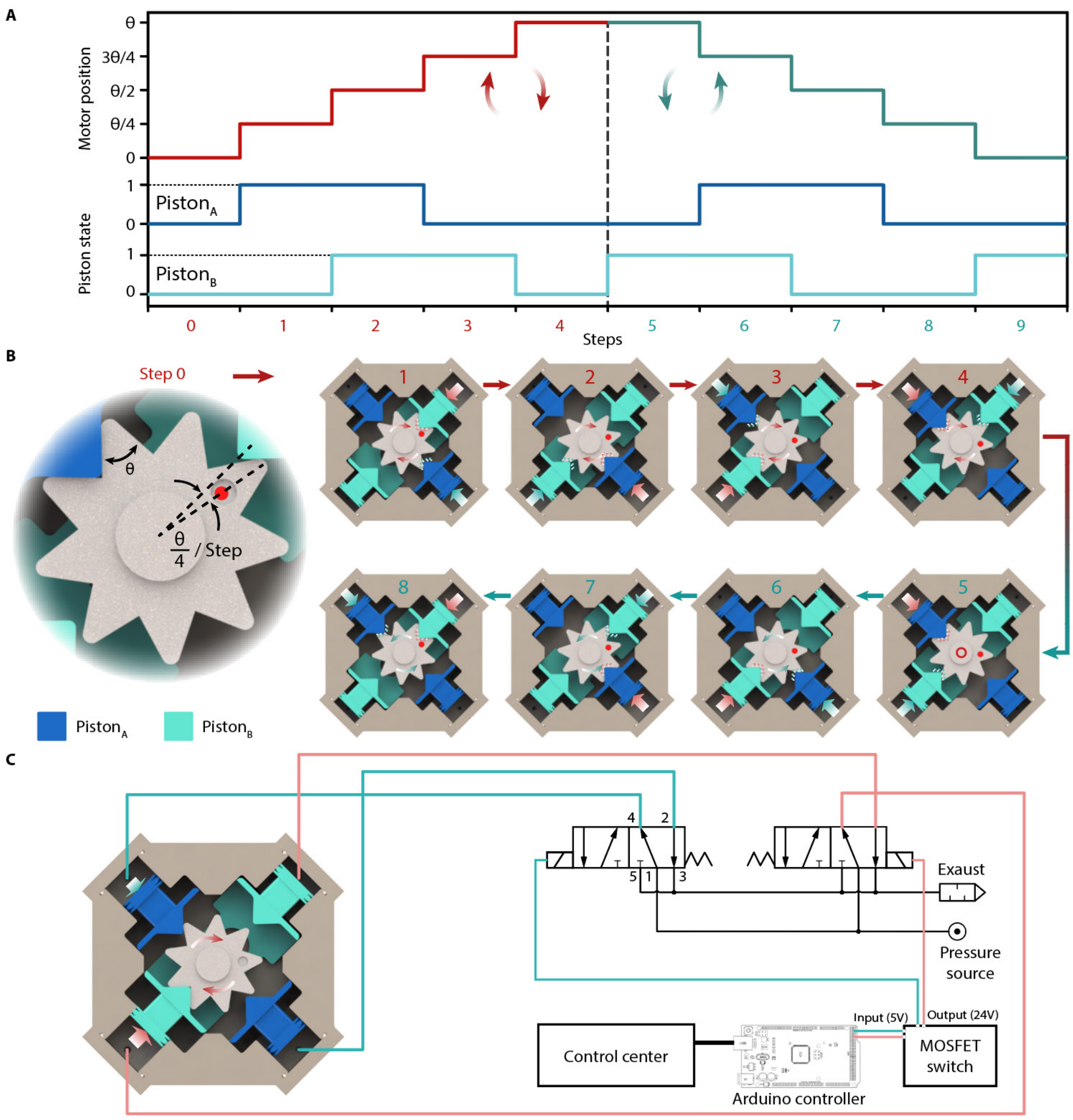

Supplementary Figure 3. Principle of the proposed stepper pneumatic actuator. (**A**) Step sequence design. Starting from 0, the sequence illustrates the movement of one tooth (θ) through four steps with CW and CCW actuation mode. Piston$_A$ transitions from state 0 to state 1 (step 1), followed by Piston$_B$ transitioning from state 0 to state 1 (step 2). Subsequently, Piston$_A$ returns to state 0 (step 3), and Piston$_B$ finally returns to state 0 (step 4). During the actuation of one piston, the other piston maintains its current state. From step 5, the motion reverses with a hysteresis effect as shown in **B**. The subsequent sequence is the inverse of steps 0-4, thereby enabling reverse rotation. (**B**) The relative positions of the pistons are shown at each step, with each step corresponding to a rotation of $\theta/4$. (**C**) The basic control framework of the stepper pneumatic actuator.

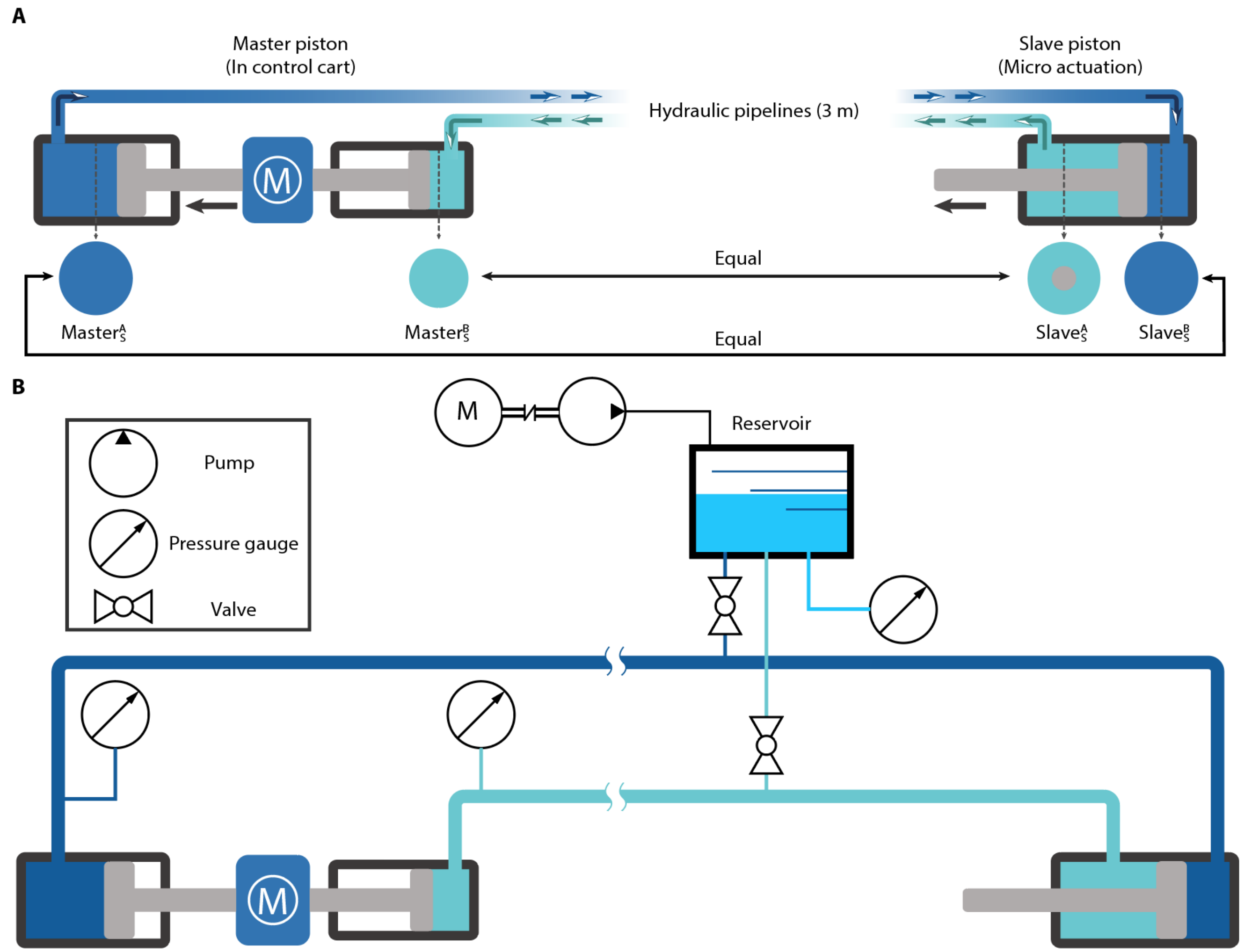

Supplementary Figure 4. Principle of the proposed hydraulic actuator. (**A**) Schematic diagram of the master-slave hydraulic actuator. Due to the incompressibility of the liquid, pistons with equal cross-sectional areas are designed for the master and slave, following the relationships: $Master_S^A = Slave_S^A$, $Master_S^B = Slave_S^B$ The master piston, activated by a motor (M), pushes the liquid to the slave piston, thereby achieving displacement equivalence. The master is housed within the control cart, which is equipped with electromagnetic shielding, enabling placement near the MR bore (<40 mT). The liquid is transmitted through a 3-m-long, high-strength hydraulic pipeline. (**B**) Schematic diagram showing the pipeline connection of hydraulic actuator with preloading system and pressure sensors.

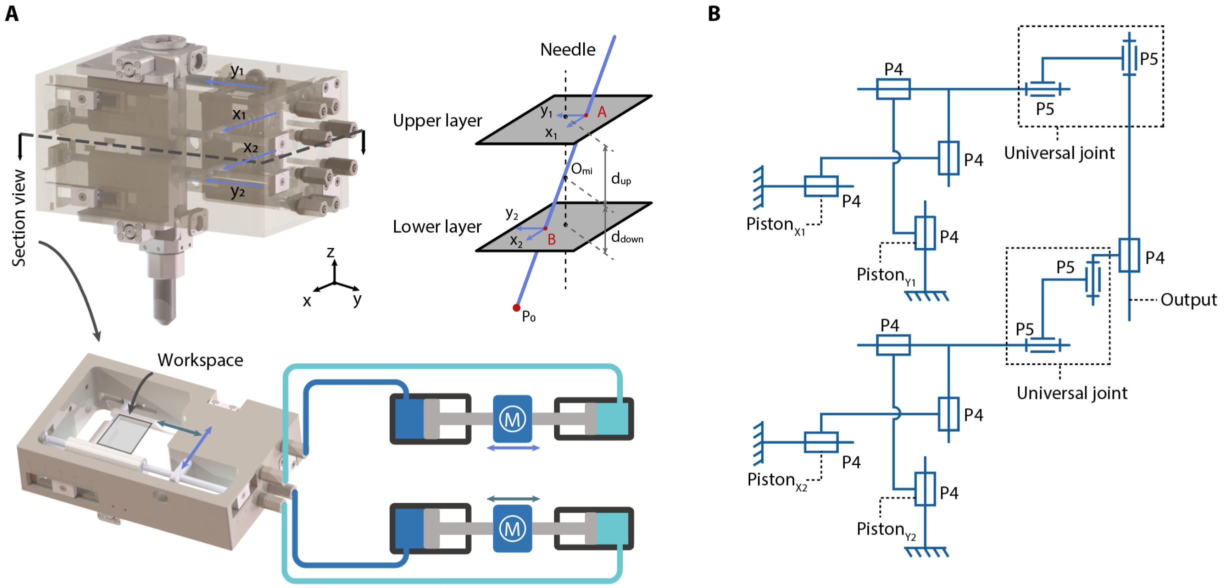

Supplementary Figure 5. Design of the 4-DoF hydraulic micro actuation system and its DoF analysis. (**A**) A parallel structure in which each plane is driven by two hydraulic pistons, enabling independent motion along the x and y axes, respectively. The workspace on each plane is a rectangle. (**B**) Constraint relationships and DoF calculation. The mechanism comprises 10 moving parts, 9 P4 joints (with rotation and translation), and 4 P5 joints (with rotation). The total DoF is calculated as $10 \times 6 - 9 \times 4 - 4 \times 5 = 4$. For the upper and lower planes, each incorporates two P5 joints configured as a universal joint, providing two rotational DoF, with the two universal joints connected by a rigid link. The upper universal joint is rigidly fixed to the link, whereas the lower universal joint is connected to the link through a cylindrical bushing (P4, sleeve joint). This configuration prevents over-constraint of the link during axial length variations.

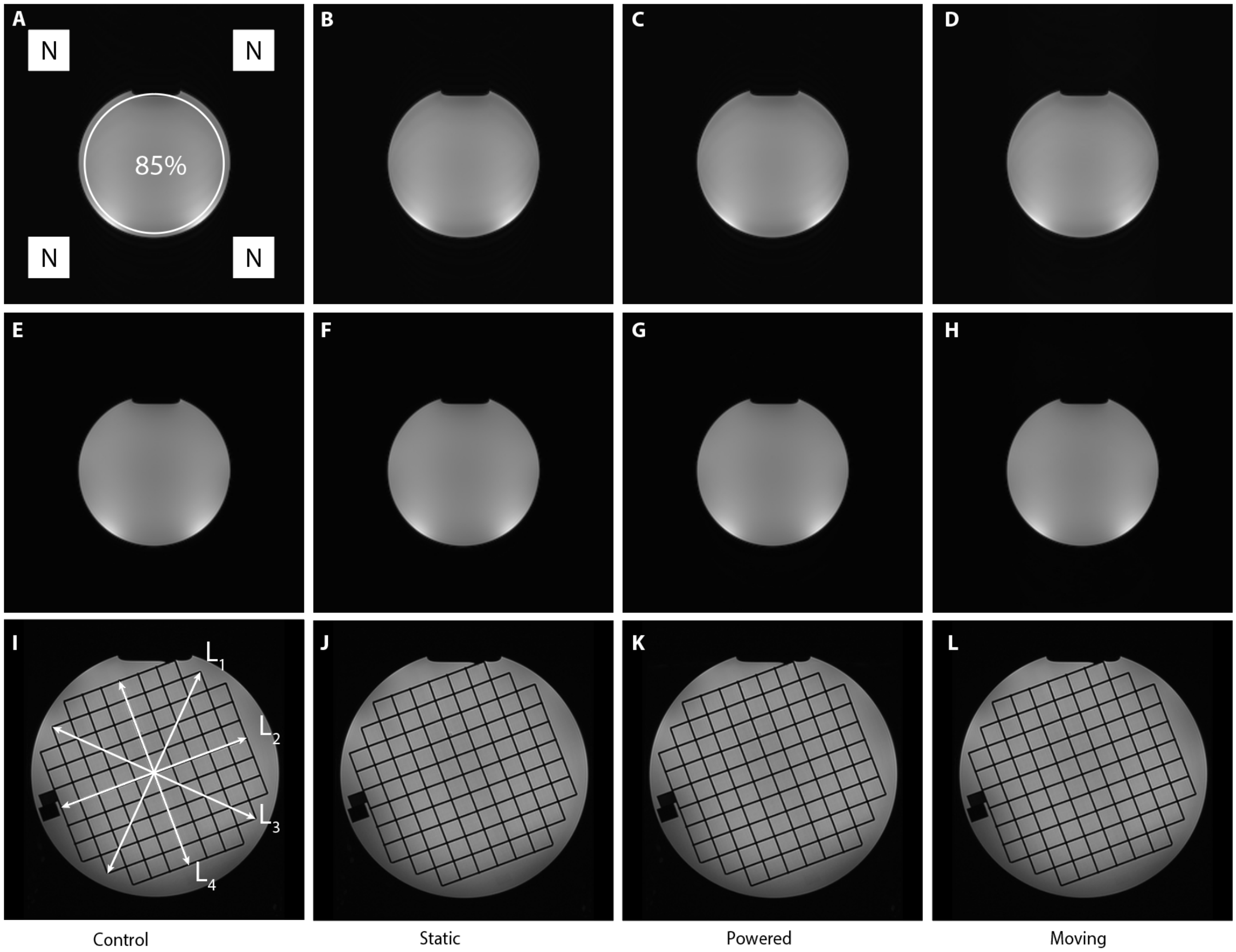

Supplementary Figure 6. MR compatibility test of the robot system. (**A**)-(**H**) SNR_{loss} test results. (**A**) Noise detection region (N) for noise testing, along with the 85% ROI for signal quantification. (**A**)-(**D**) MRI images under the T1-weighted sequence. (**E**)-(**H**) MRI images under the T2-weighted sequence. (**I**)-(**L**) Geometric distortion test images under the T2-weighted sequence. (**I**) The method for measuring the lengths L_1, L_2, L_3 and L_4.

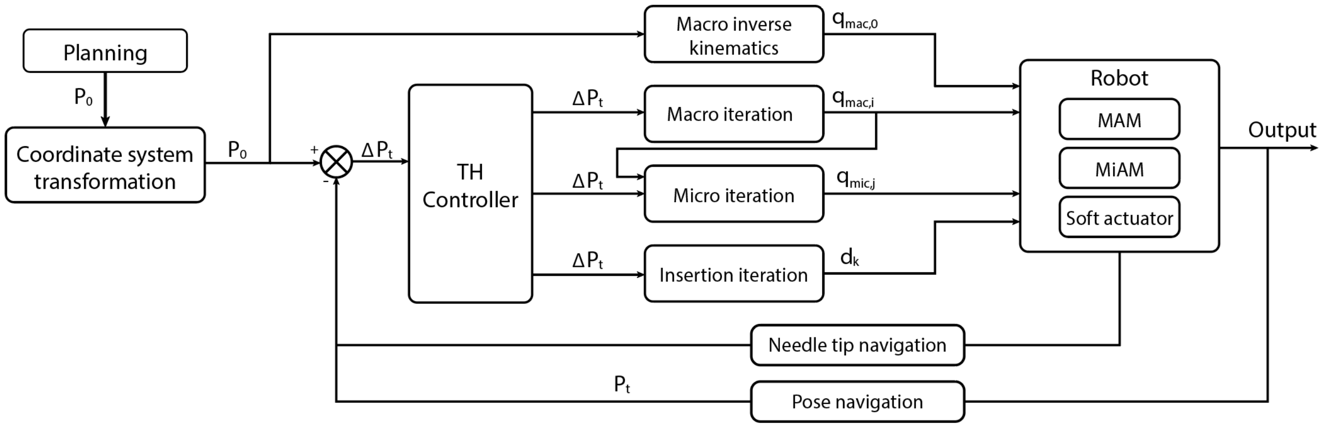

Supplementary Figure 7. Macro-micro task hierarchical control framework.

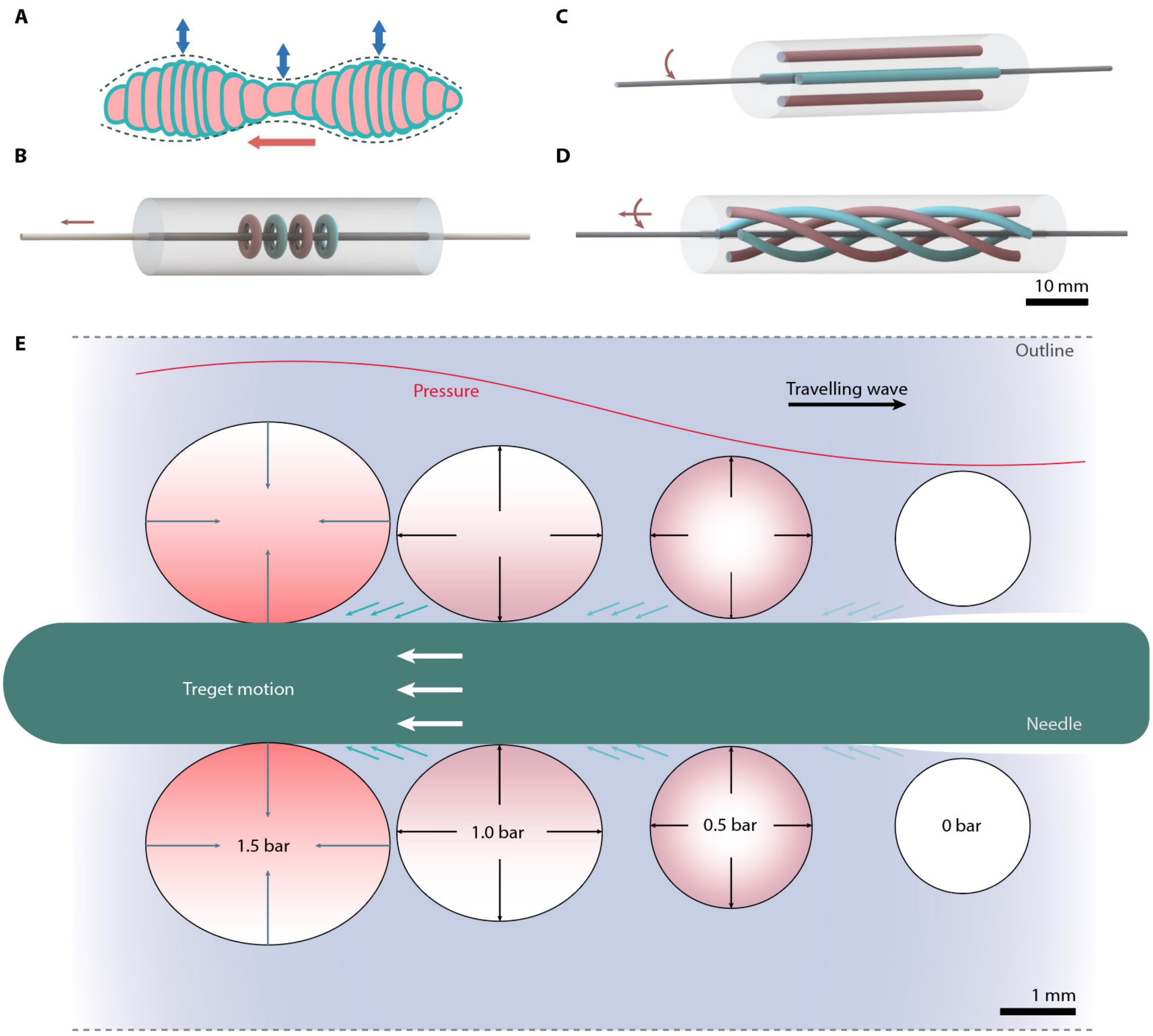

Supplementary Figure 8. Bionic and travelling wave principle of the bio-inspired soft actuator. (**A**) A worm achieves linear propulsion through traveling waves generated by sequential radial expansion and contraction of body segments. (**B**) The annular cavity configuration generates linear motion. (**C**) Linear cavity facilitates rotational motion around the axis. (**D**) Helical cavity enables simultaneous linear-rotational motion. (**E**) Schematic diagram of elastic deformation during traveling wave propagation. Pressure variations in different chambers enable partial contact and separation in specific regions, where the expansion elastic force in the non-contact state propels the elastic structure in the contact state to drive the displacement of the needle.

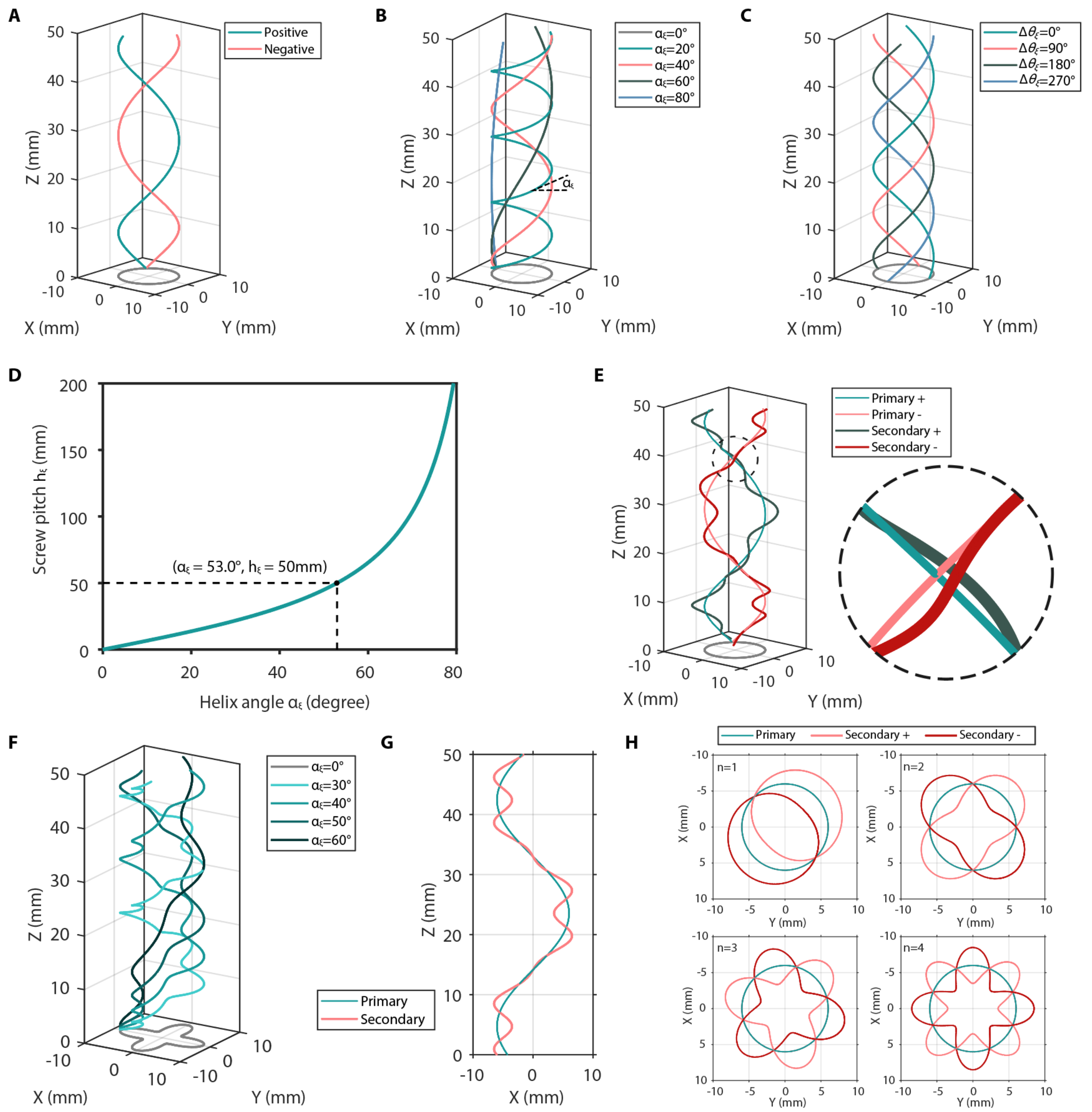

Supplementary Figure 9. Geometric characterization of the helical chamber architectures. (**A**) Fundamental helical curves with bidirectional propagation properties. (**B**) Helical architectures with parametric variations in helix angle (α_ξ=0°, 20°, 40°, 60°, 80°). (**C**) Helical configurations with different initial phases ($\Delta\theta_\xi$=0°, 90°, 180°, 270°). (**D**) Quantitative correlation between pitch (h_ξ) and helix angle (α_ξ). (**E**) Structural decomposition methodology of quadratic helical curves. (**F**) Quadratic helical configurations with different helix angles (α_ξ=0°, 30°, 40°, 50°, 60°). (**G**) Comparative analysis of primary (n=0) and quadratic (n=4) helical topologies. (**H**) Angular wave frequencies with different parameters (n=1, 2, 3, 4).

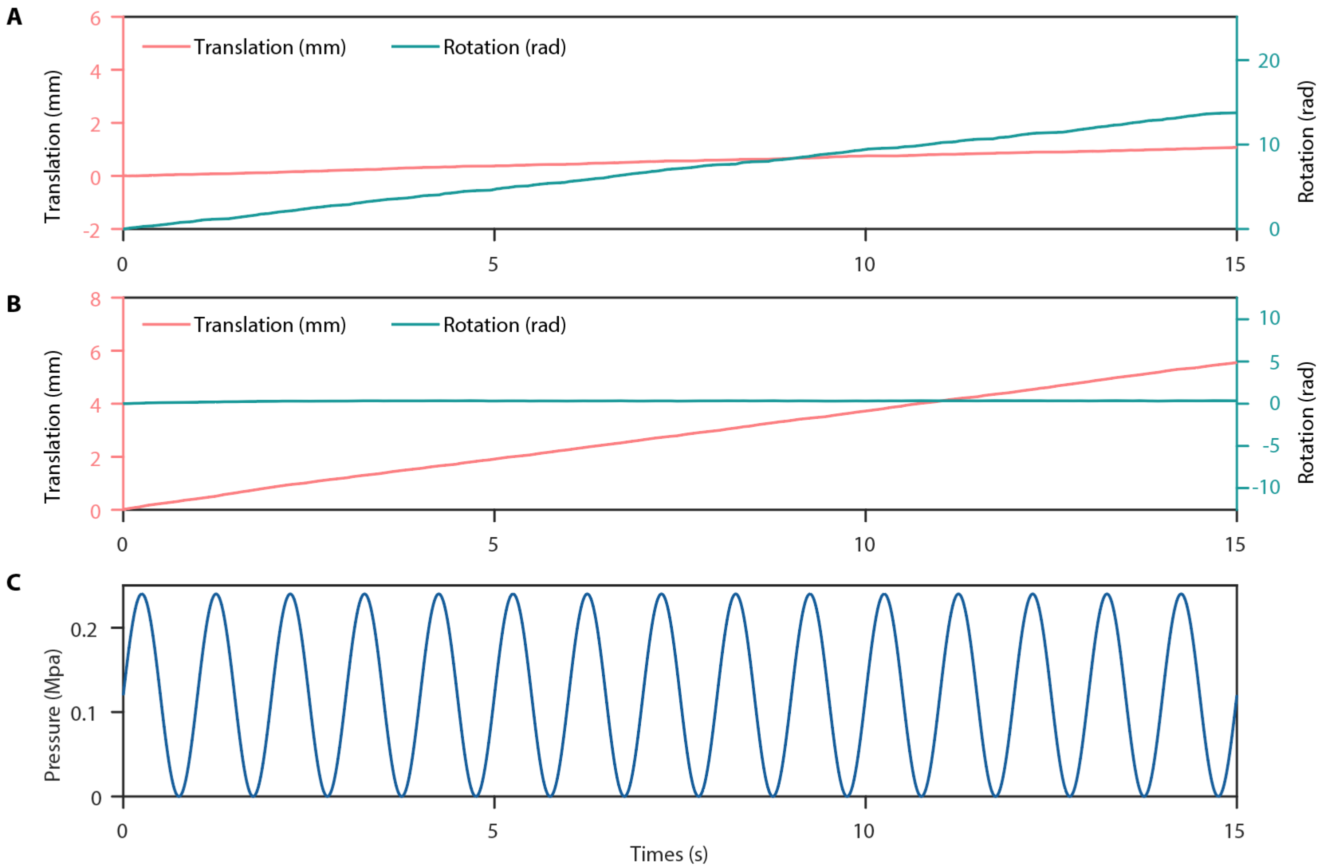

Supplementary Figure 10. FEA results of decoupling mode performance. (**A**) Output profiles of displacement and rotation in rotational mode. (**B**) Output profiles of displacement and rotation in translational mode. (**C**) Traveling wave pressure variations within individual chambers.

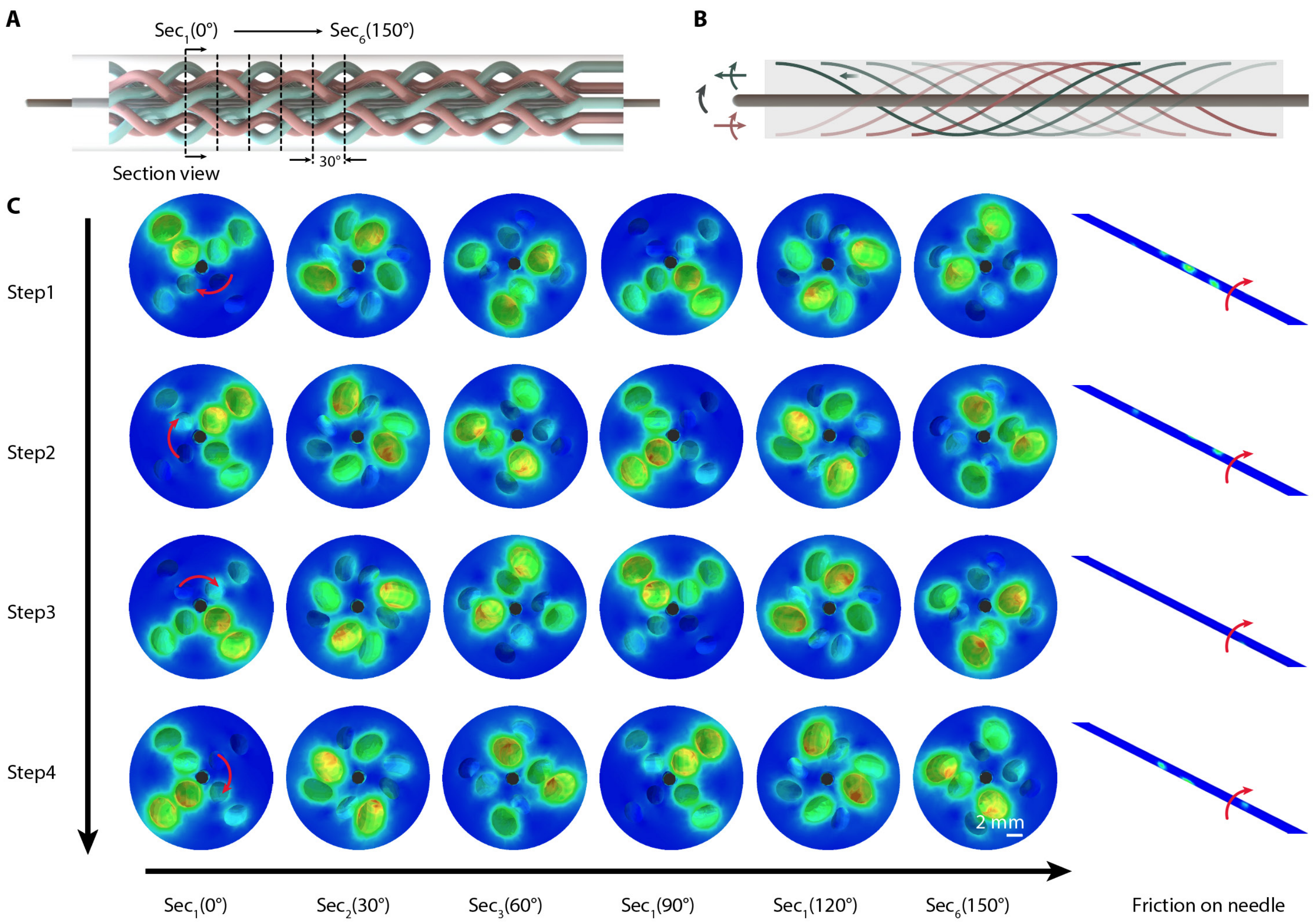

Supplementary Figure 11. FEA results of the bio-inspired soft actuator in the independent rotation mode. (**A**) The cross-sectional positions in the structure are illustrated at 30° increments, covering phase angles from 0° to 150°. (**B**) Travelling wave input for the independent rotational mode. (**C**) A full motion cycle is divided into four steps, with the cross-sectional deformation profiles depicted. The horizontal axis corresponds to sequentially arranged phase angles, while the right column shows contact stress distributions on the needle surface for each step.

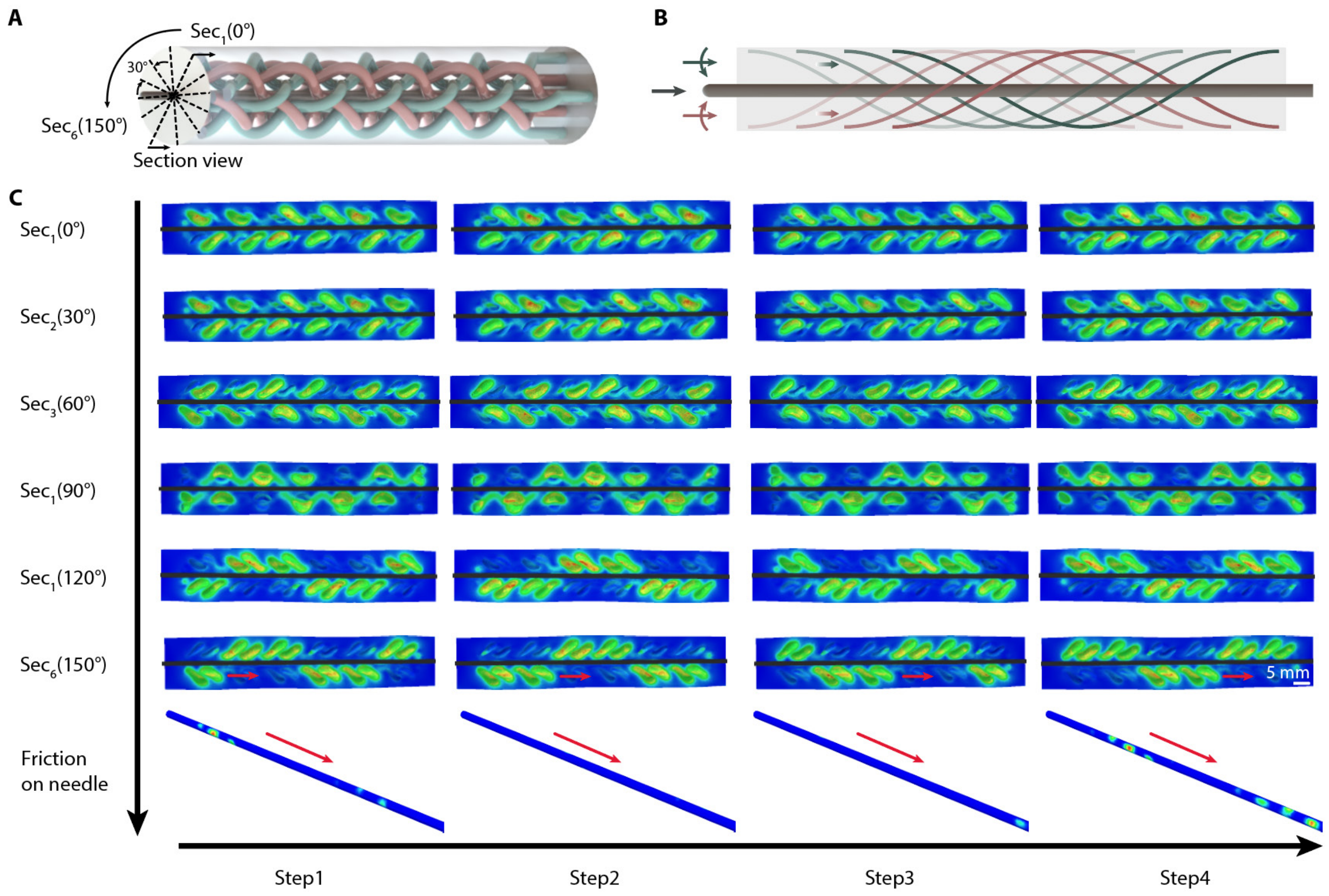

Supplementary Figure 12. FEA results of the bio-inspired soft actuator in the independent translation mode. (**A**) The cross-sectional positions in the structure are illustrated at 30° increments, covering phase angles from 0° to 150°. (**B**) Travelling wave input for the independent translational mode. (**C**) A full motion cycle is divided into four steps, with the cross-sectional deformation profiles depicted. The vertical axis corresponds to sequentially arranged phase angles, while the bottom row shows contact stress distributions (contour maps) on the needle surface for each step.

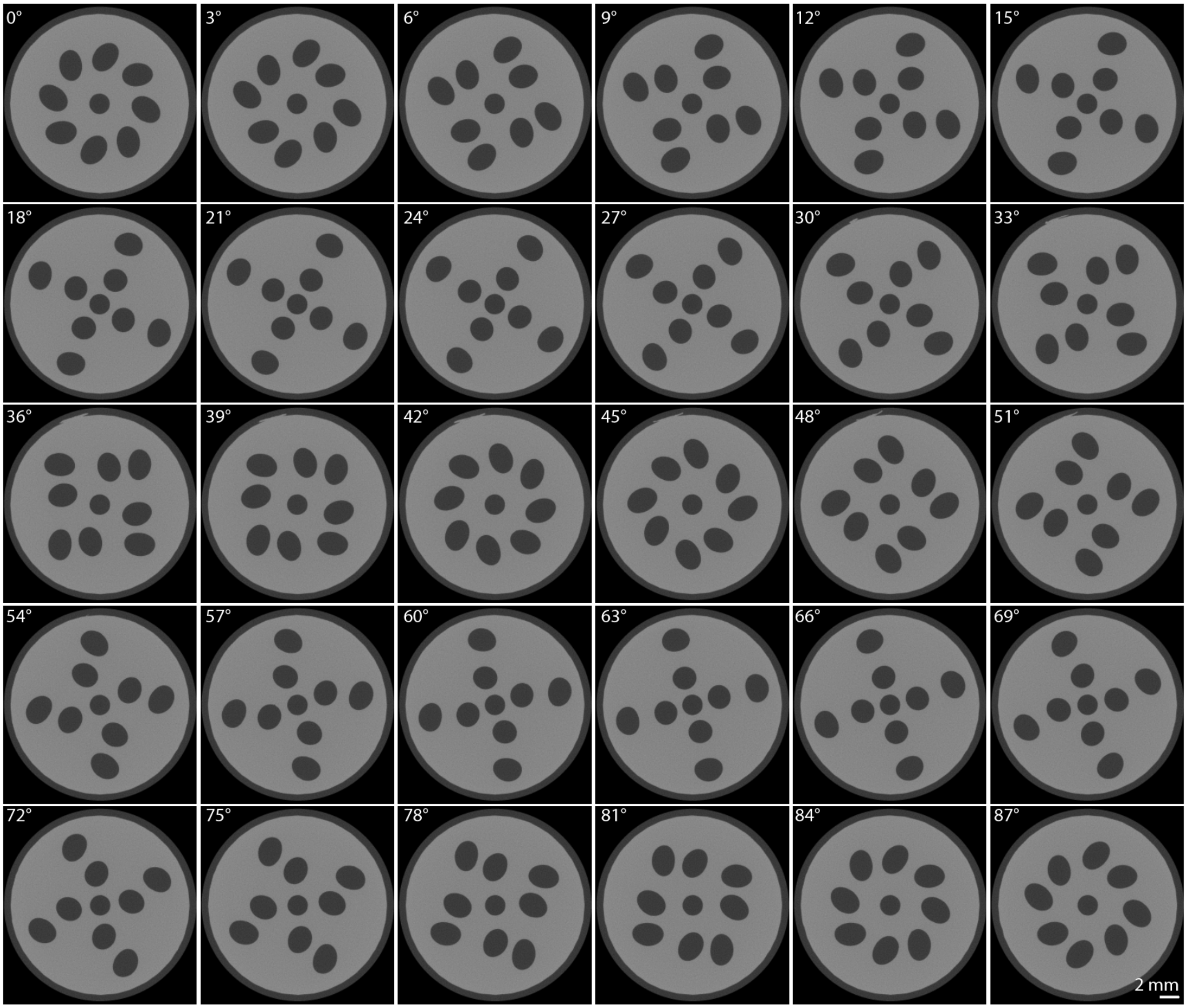

Supplementary Figure 13. Micro-CT imaging of the bio-inspired soft actuator. Cross-sectional views are displayed at 3° phase increments, capturing structural interface variations from 0° to 87°. Owing to inherent structural symmetry, the regions from 90° to 360° would exhibit analogous morphological characteristics. The micro-CT images demonstrate chamber independence, with CW and CCW helical chambers maintaining uniform spacing and gradual pitch variation.

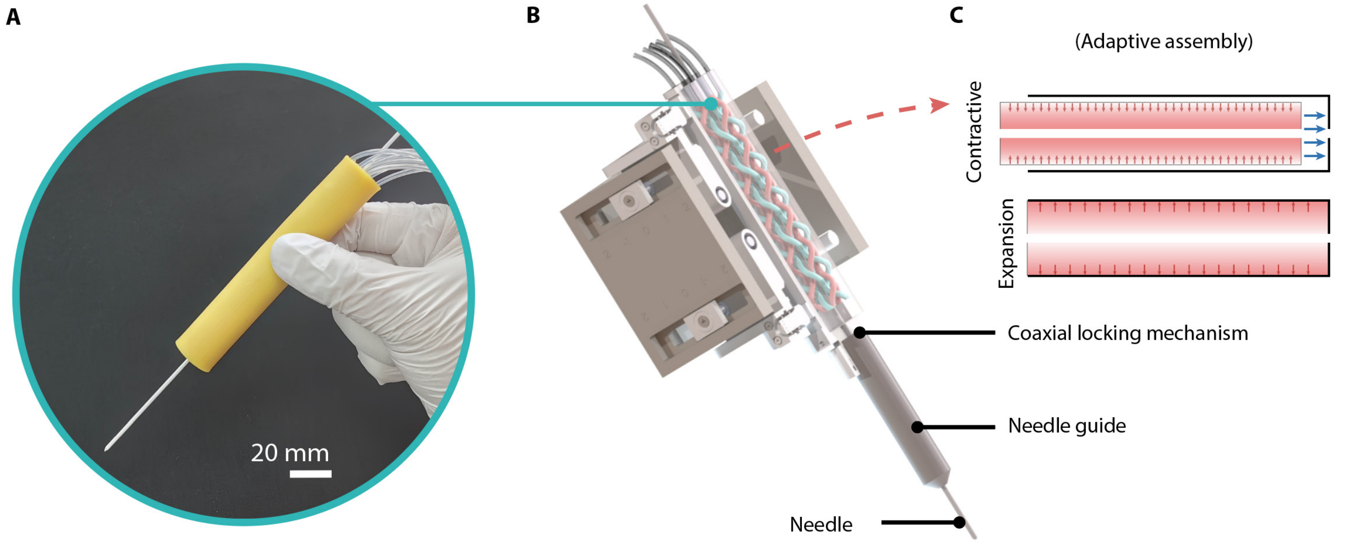

Supplementary Figure 14. Assembly of the soft actuator and needle guide. (**A**) Prototype of the bio-inspired soft actuator with the ceramic needle. (**B**) Cross-sectional view of micro-actuation integrated soft actuator and needle guide. A needle guide for MRI guidance is coaxially aligned with the actuator to provide needle trajectory guidance, while the ceramic needle is manually inserted through the actuator and the needle guide. (**C**) Adaptive assembly method for soft actuators. Subjected to negative pressure pre-compression, the soft actuator achieves volumetric contraction to facilitate insertion into the micro-platform's central orifice. Subsequent pressure restoration triggers radial expansion, establishing self-locking between the actuator and platform interface.

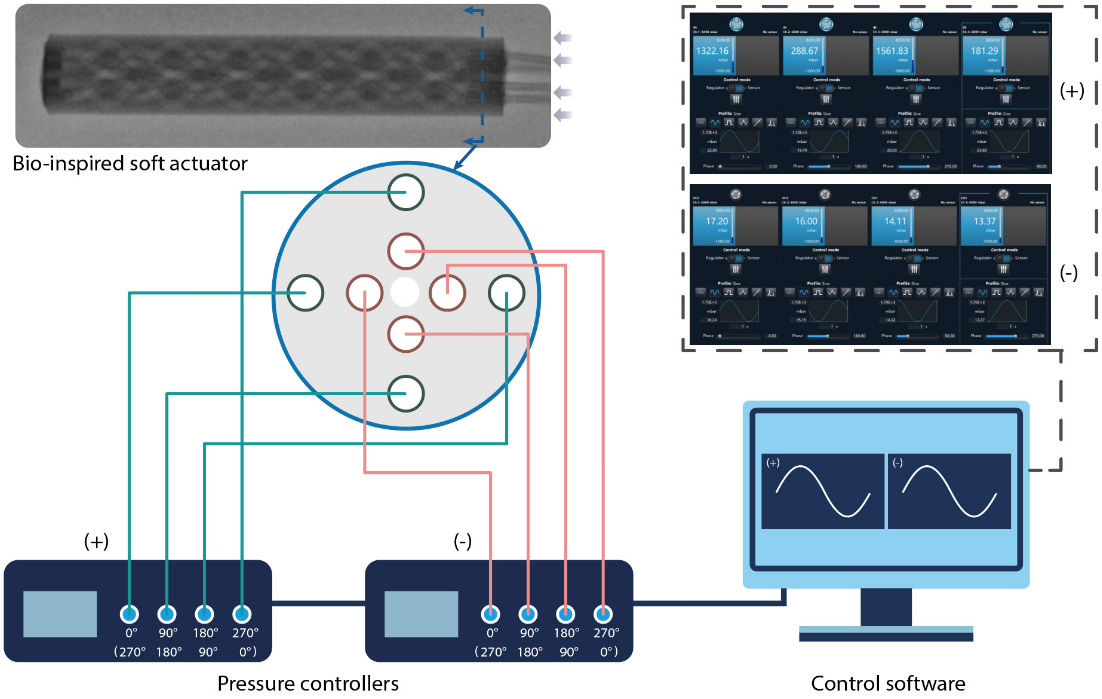

Supplementary Figure 15. Control system of the bio-inspired soft actuator. The pressure control system for the soft actuator comprises a host PC and a multi-channel pressure controller. Each chamber possesses independent pressure-regulation capability. Mounted on the robotic micro-platform within the MRI bore, the soft actuator connects via pneumatic transmission lines. The pressure controller resides in a shielded enclosure outside the scanning room to ensure MR compatibility.

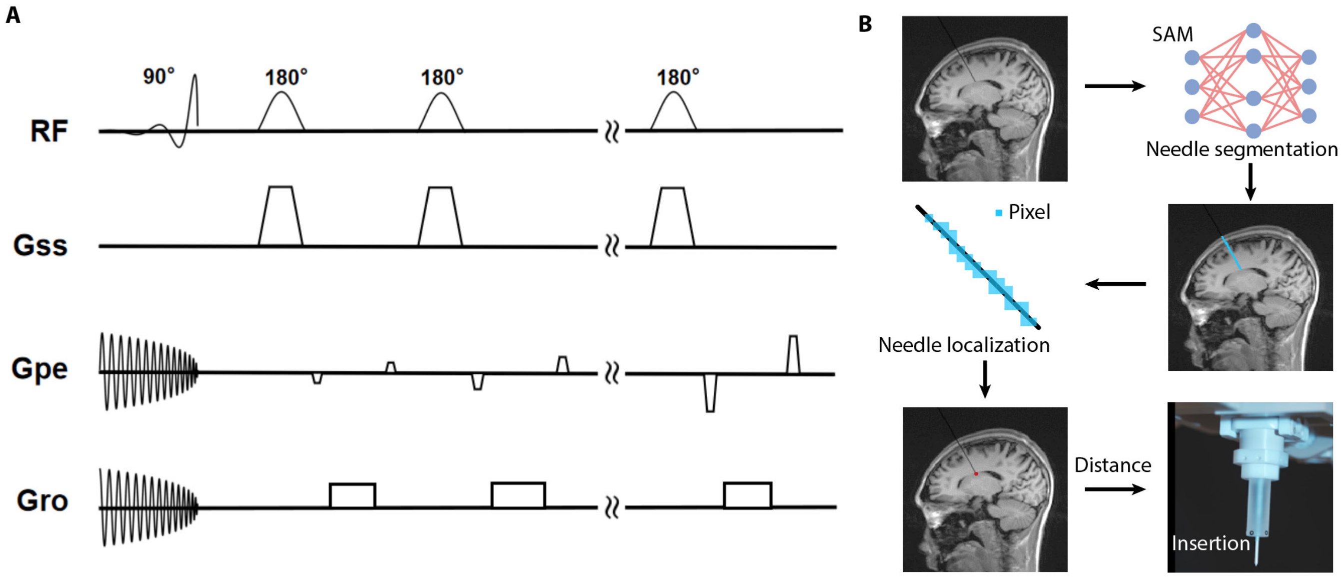

Supplementary Figure 16. Illustrations of 2D rFOV FSE MRI and needle tracking. (**A**) The sequence diagram of 2D FSE with reduced FOV. (**B**) Needle tracking workflow. The needle is initially segmented via SAM with a bounding box prompt, followed by direction and tip localization through a least-squares fitting algorithm.

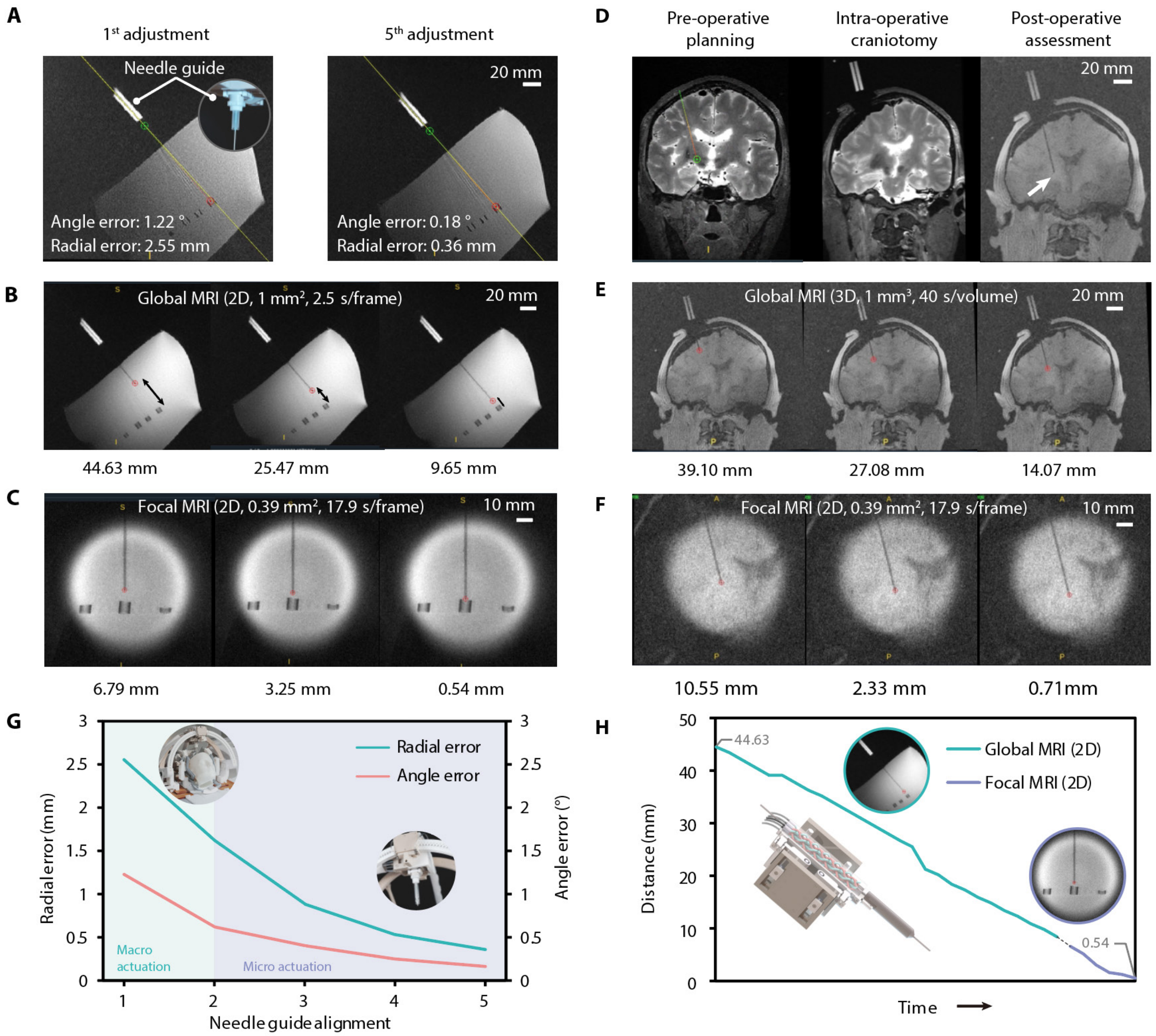

Supplementary Figure 17. Results of phantom and cadaveric studies. (**A**) The 1st and 5th adjustments of the needle guide alignment. (**B**) 2D global MRI during robotic intervention in the phantom experiment. (**C**) 2D focal MRI during robotic intervention in the phantom experiment. (**D**) A comparison of pre-, intra-, and post-operative MRI of the cadaveric experiment. (**E**) 3D global MRI during robotic intervention in the cadaveric experiment. The central slice was chosen for display. (**F**) 2D focal MRI during robotic intervention in the cadaveric experiment. (**G**) The process of needle guide alignment in the phantom experiment. (**H**) Continuous distance monitoring between needle tip and target during robotic advancement in the phantom experiment.

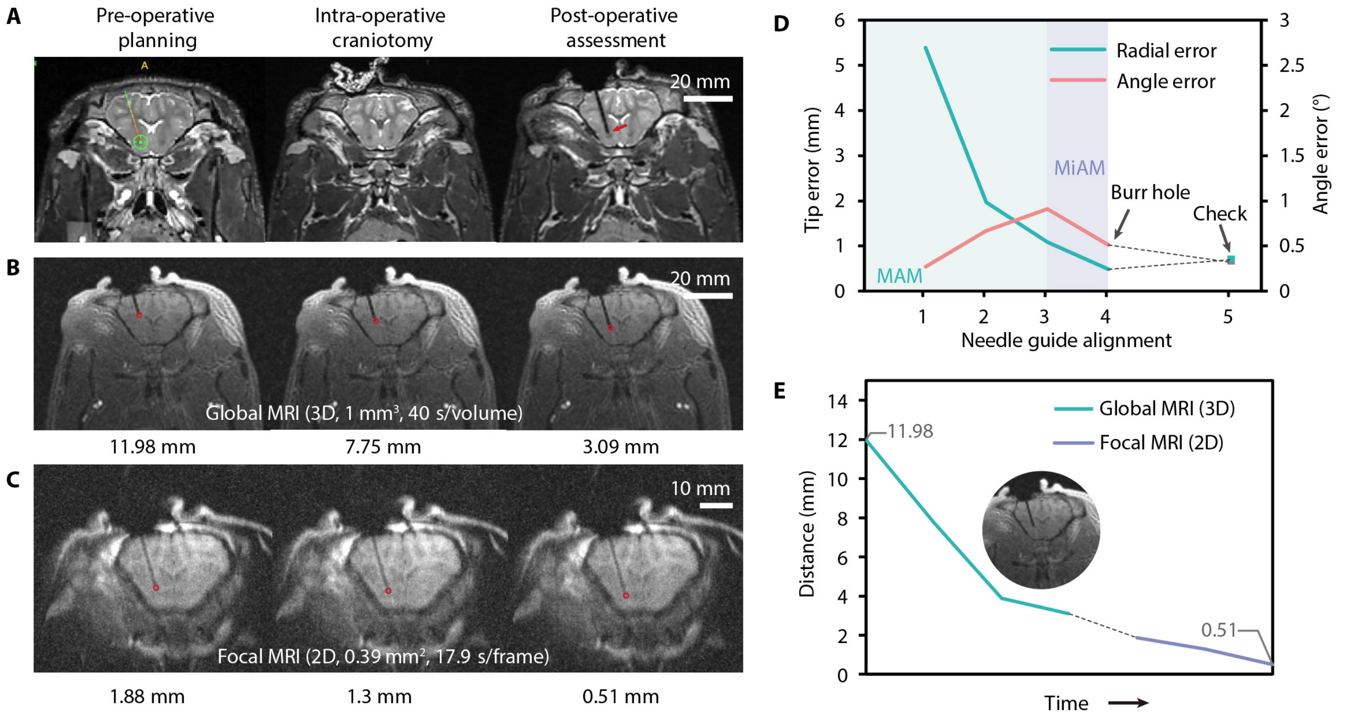

Supplementary Figure 18. Results of in vivo study. (**A**) A comparison of pre-, intra-, and post-operative MRI of the study. (**B**) 3D global MRI during robotic intervention in the study. The central slice was chosen for display. (**C**) 2D focal MRI during robotic intervention in the study. (**D**) The process of needle guide alignment in the study. (**E**) Continuous distance monitoring between needle tip and target during robotic advancement in the study.

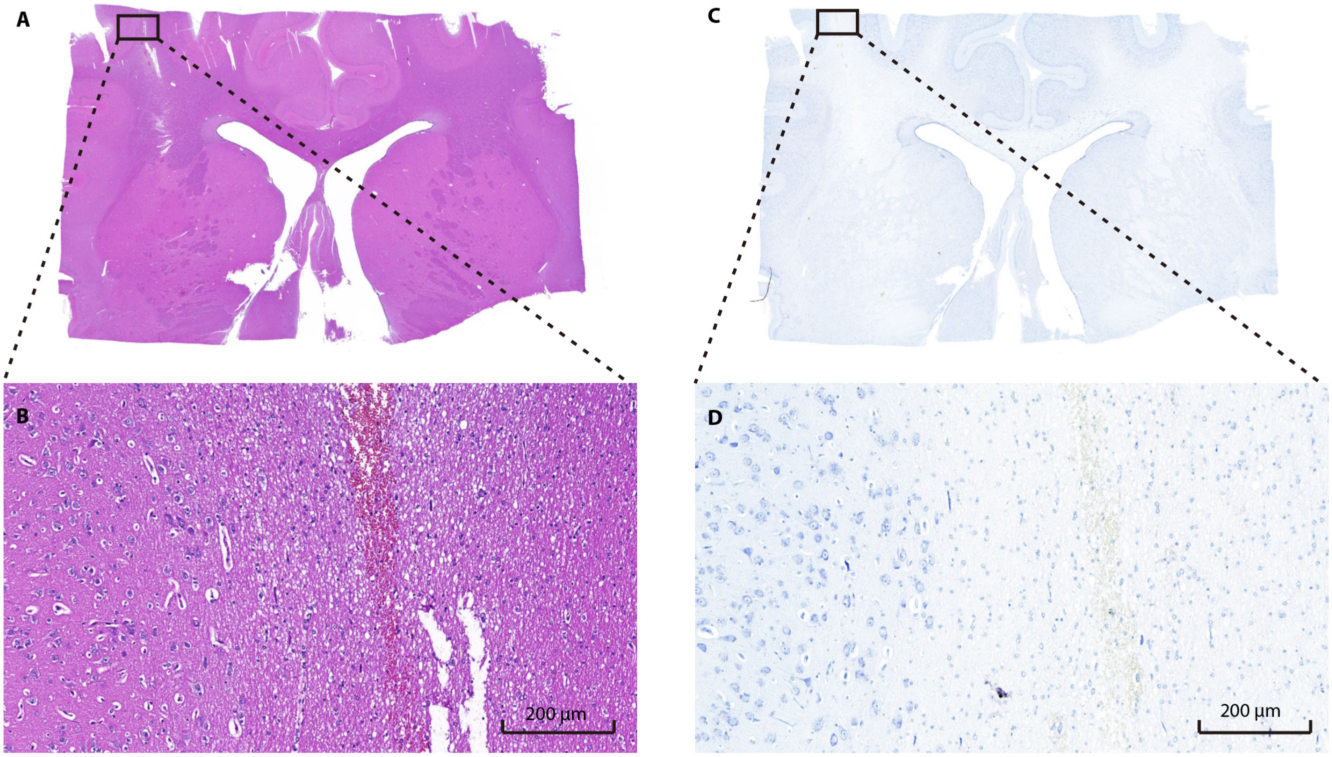

Supplementary Figure 19. Results of pathological section staining. (**A**, **B**) H&E staining image of the insertion site. (**C**, **D**) Nissl staining image of the insertion site.

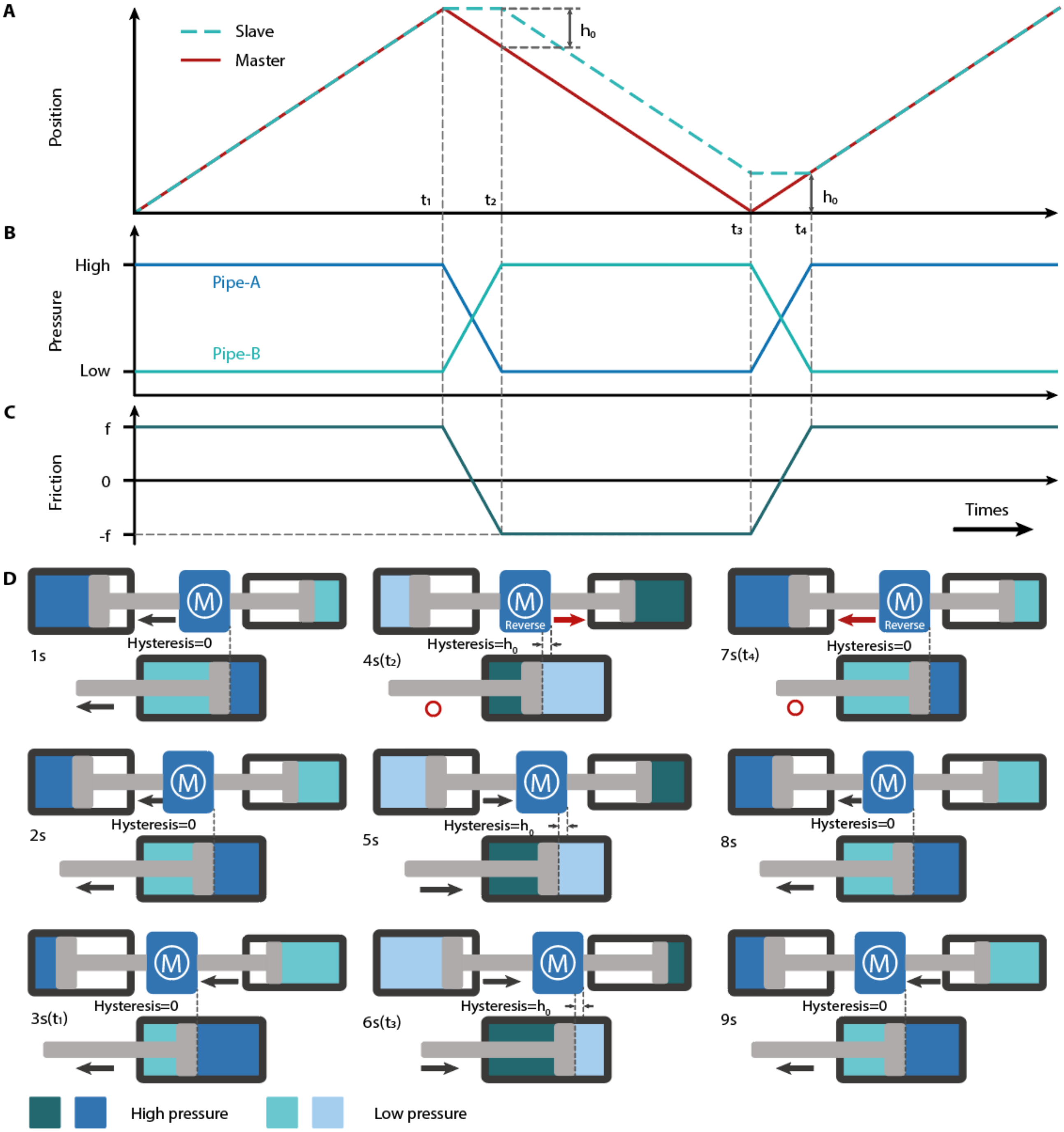

Supplementary Figure 20. Master-slave coupling hydraulic actuation principle. (**A**) Displacement variation curves of master and slave piston. (**B**) Pressure variation curves of pipeline A and B. (**C**) Friction force variation curve of slave piston. (**D**) Displacement relationship during the hydraulic transmission process. At 4 s (t_2) and 7 s (t_4), the hysteresis effect during the reverse process is observed, where the pressures in the two pipelines (green and blue) transition to distinct states (dark color for high pressure, and light color for low pressure), while the slave piston position remains unchanged. At 2 s and 8 s, the slave piston reaches equivalent positions, demonstrating the unidirectional absolute positioning capability of the hydraulic actuator.

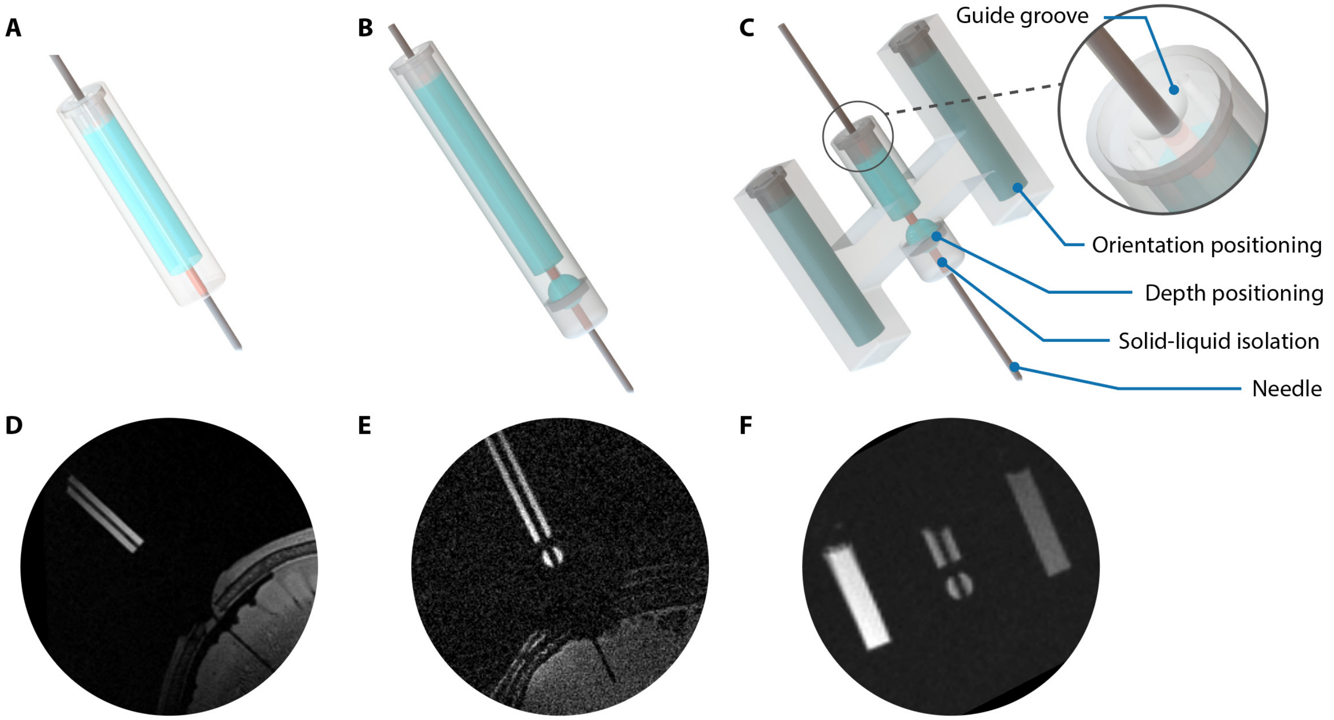

Supplementary Figure 21. Design of MR-compatible imaging needle guides. (**A**) Short needle guide for directional positioning imaging. (**B**) Long needle guide for directional and depth positioning imaging. (**C**) Short needle guide for directional and depth positioning imaging. (**D-F**) MRI imaging of these needle guides.

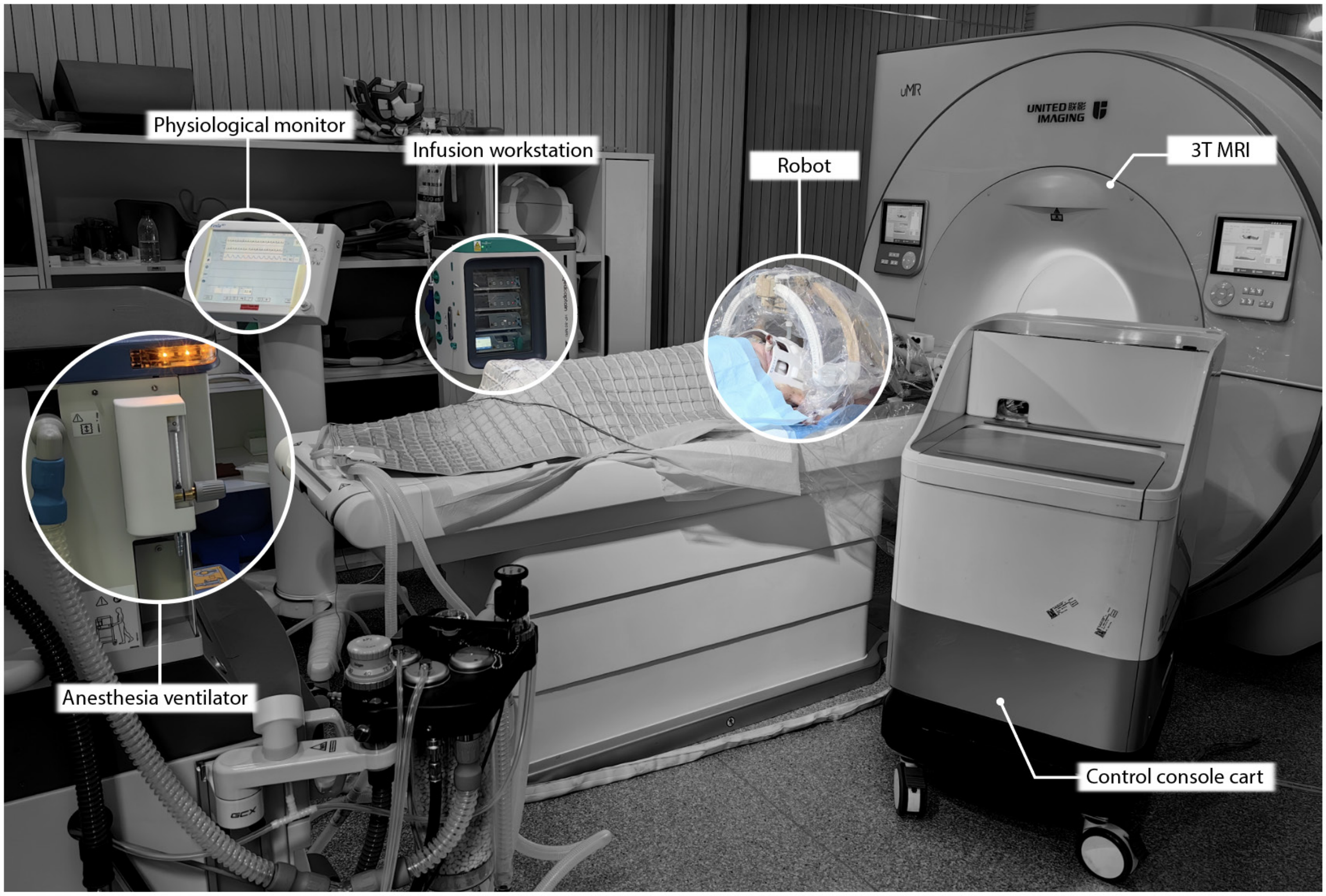

Supplementary Figure 22. In vivo experimental setup. The setup comprises: a porcine subject, robot, control console cart, physiological monitor, anesthesia ventilator, infusion workstation, and 3T MRI scanner.

Supplementary Table

Supplementary Table 1. Comparison of the MRI-guided robotic stereotactic neuro-intervention systems.

Intraoperative MRI guided system for neurosurgery	Stereotactic	Needle insertion	Actuation	MR compatibility (SNR_{loss})	Workspace	Targeting accuracy (mm)	Interactive feedback	Ref.
SYMBIS/NeuroArm*	8-DOF (R)	1-DOF (R)	Piezoelectric	NP	D-H Table	NP	NA	(*14*)
NeuroBlate system*	3-DOF (M)	2-DOF (R)	NA	NP	±58°	1.57± 0.21 (P)	MRI-T	(*16*)
Chen *et al.*	2-DOF (M)	3-DOF (R)	Pneumatic	4.9%	NP	1.26±1.22 (P)	MRI-FB	(*37*)
Fischer *et al.*	8-DOF (R)	3-DOF (R)	Piezoelectric	10.3%	Transverse: ±45° Sagittal: 0~90°	1.45±0.66 (P)	MRI-FB	(*19*), (*20*)
Stoianovici *et al.*	3-DOF (M) +3-DOF (R)	M	Pneumatic	1%	Transverse: ±40° Sagittal: 30~-50°	1.55± 0.81 (P)	NA	(*21*), (*22*)
Kwok *et al.*	3-DOF (M) +2-DOF (R)	M	Hydraulic	2.7%	Coarse: ±30° Fine: ±5°	1.7 (P) 2.2 (C)	NA	(*23*), (*24*)
**This paper**	**8-DOF (R)**	**2-DOF (R)**	**Hybrid hydraulic-pneumatic**	**2.3%**	**Transverse: ±60° Sagittal: 20°~-60°**	**0.39±0.12 (P) 0.68±0.13 (C) 0.14 (V)**	**MRI-P**	

Notes: *Commercial product. M: manual, R: Robotic. NA and NP are abbreviations for not applicable and not provided, respectively. P: phantom results, C: cadaver results, V: in vivo results, MRI-FB: MRI feedback, MRI-T: MR-based temperature feedback, MRI-P: MR-based position feedback.

Supplementary Movies

Movie S1. Overview video of this work. The traditional procedure of stereotactic neurosurgery and its associated challenges are introduced. The methods proposed in this work are organized according to the three stages of stereotactic neurosurgery: pre-operative, intra-operative, and post-operative. Experimental results from both phantom and cadaveric studies are demonstrated. Finally, the solutions proposed in this work to address clinical challenges are summarized, highlighting the three main contributions.

Movie S2. Hydraulic micro motion. This video demonstrates the motion capabilities of the MiAM system in all 4-DoF. Specifically, it shows the independent motion of joints x_1 and y_1, as well as the coordinated motion of x_1-x_2, and y_1-y_2 pairs.

Movie S3. Performance characterization of the bio-inspired soft actuator. The video demonstrates the basic helical motion of the CW chamber and CCW chamber, which is a synchronized rotational and translational motion. Due to the opposing structures of CW and CCW chambers, the output translational direction is inverted without altering the rotational orientation. The video further demonstrates the independent control of needle rotation and translation, along with time-dependent position and angle curves obtained through camera-based tracking, as well as corresponding FEA simulations.

Movie S4. Repeatability test of the micro actuation system. This video demonstrates the repetitive bidirectional motion of the MiAM system, using a microscale marker tracked by a high-resolution camera to measure the needle tip position and evaluate positional repeatability.

Movie S5. Interactive needle tracking in MRI. The video demonstrates the intervention process in simulated human brain and ex vivo porcine brain tissue, including original MRI images, needle segmentation results (blue mask), and needle tip localization (red dot).

Movie S6. Global-focal MRI-guided robotic intervention for phantom study. During robotic intervention in phantom, global-focal MRI captures images continuously, while the SAM-based needle tracking algorithm monitors the needle tip (red crosshair). The live chart displays the distance between the needle tip and the target.

Movie S7. Global-focal MRI-guided robotic intervention for cadaveric study. During robotic intervention in cadaver, global-focal MRI captures images continuously, while the SAM-based needle tracking algorithm monitors the needle tip (red crosshair). The live chart displays the distance from the needle tip to the target.
